# The Adipokinetic Peptides of Hemiptera: Structure, Function, and Evolutionary Trends

**DOI:** 10.3389/finsc.2022.891615

**Published:** 2022-06-15

**Authors:** Gerd Gäde, Heather G. Marco

**Affiliations:** Department of Biological Sciences, University of Cape Town, Rondebosch, South Africa

**Keywords:** Hemiptera, true bugs, phylogeny of Hemiptera, insect flight, lipid metabolism, regulation by adipokinetic hormone (AKH), AKH primary structure, molecular evolution of hemipteran AKHs

## Abstract

The Hemiptera comprise the most species-rich order of the hemimetabolous insects. Members of a number of superfamilies, most notably especially the more basal ones such as white flies, psyllids and aphids, belong to the most destructive agricultural insects known worldwide. At the other end of the phylogenetic tree are hemipterans that are notorious medical pests (e.g. kissing bugs). Most of the hemipteran species are good flyers, and lipid oxidation plays a pivotal role to power the contraction of flight muscles and, in aquatic water bugs, also deliver the ATP for the extensive swimming action of the leg muscles. Mobilization of stored lipids (mostly triacylglycerols in the fat body) to circulating diacylglycerols in the hemolymph is regulated by a set of small neuropeptides, the adipokinetic hormones (AKHs). We searched the literature and publicly available databases of transcriptomes and genomes to present here AKH sequences from 191 hemipteran species. Only few of these peptides were sequenced *via* Edman degradation or mass spectrometry, and even fewer were characterized with molecular biology methods; thus, the majority of the AKHs we have identified by bioinformatics are merely predicted sequences at this stage. Nonetheless, a total of 42 AKH primary sequences are assigned to Hemiptera. About 50% of these structures occur also in other insect orders, while the remaining 50% are currently unique for Hemiptera. We find 9 novel AKHs not shown to be synthesized before in any insect. Most of the hemipteran AKHs are octapeptides (28) but there is an impressive number of decapeptides (12) compared to other speciose orders such as Diptera and Lepidoptera. We attempt to construct a hypothetical molecular peptide evolution of hemipteran AKHs and find quite a bit of overlapping with current phylogenetic ideas of the Hemiptera. Lastly, we discuss the possibility to use the sequence of the aphid AKH as lead peptide for the research into a peptide mimetic fulfilling criteria of a green insecticide.

## 1 Introduction: Scope and Intent of this Review

This review deals mainly with the sequenced structures of the adipokinetic peptides known from various hemipteran families, and the role of these peptides on lipid metabolism where known. Since we look at these sequences also from an evolutionary point of view, we first introduce the reader with a short account into the world of Hemiptera by reporting on the phylogenetic relationships within this insect order and biological lifestyle facts of select species that will be dealt with later. We introduce the adipokinetic hormone (AKH) family to set the scene for our later dealings with the different insects and the function the AKH may have in those species, especially during locomotory activity, such as flight and swimming. We mainly concentrate on the metabolic function of AKH but, where appropriate, we digress and mention other functional aspects too. Thus, the review covers fundamental concepts and issues of endocrinology, physiology and phylogeny, as well as revealing current research gaps in the topic of Hemipteran AKHs.

**Figure 1:**
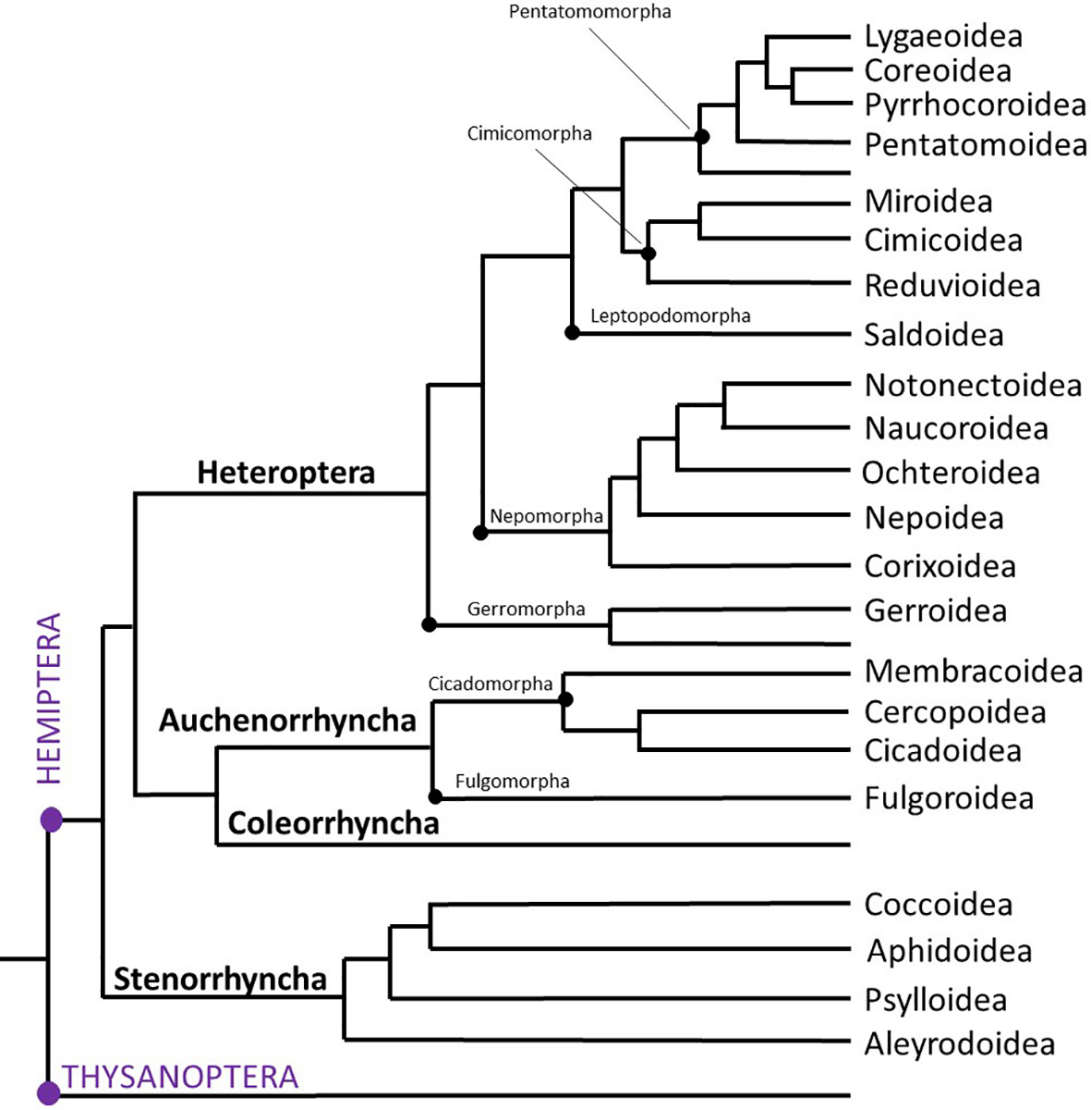
Evolutionary tree of Hemiptera. Adapted from Johnson et al., 2018 (3), this condensed tree reflects only the superfamilies dealt with in the current study. Orders are indicated in purple CAPITAL LETTERS (left); Suborders are in bold letters (left); Infraorders are shown with a black dot and by name on the tree, while superfamilies are indicated on the right of the tree.

Starting off with a historical account of the discovery of the AKHs in Hemiptera, the review will deal with the evolutionary groups of Hemiptera as outlined in **Table 1** and **Figure 1**. Once the various AKHs present in the hemipteran families are introduced, we evaluate the sequence information under the following criteria: novel structures unique for Hemiptera, molecular evolution and phylogenetic evolution. The review will not discuss the different schools of thought pertaining to the phylogeny of hemipteroid insects but will evaluate whether the AKH sequence data may enhance the current understanding of Hemiptera phylogeny. We explore the possible molecular evolution of hemipteran AKHs from the array of sequences available to date and finally, we assess/theorize on the potential of AKH analogs in the development of a so-called green insecticide in the field of insect pest control against certain very devastating hemipterans.

**Table 1:** Biodiversity of AKH peptides in Hemiptera: primary structure and calculated protonated mass.

Higher Taxonomy	Family	Species	AKH	Sequence	[M+H]^+^	Reference/Accession No.
Sternorrhyncha, Aleyrodoidea	Aleyrodidae	*Bemisia tabaci*	Galyu-AKH	pEVNFSPTWa	960.4574	(1)
		*Trialeurodes vaporariorum*	Galyu-AKH	pEVNFSPTWa	960.4574	VMOF01000220
Sternorrhyncha, Psylloidea	Psyllidae	*Diaphorina citri*	Olisa-AKH	pEVNFSPNWGGa	1087.4956	(2)
		*Heteropsylla cubana*	Olisa-AKH	pEVNFSPNWGGa	1087.4956	GCXB01018433 (3);
	Aphalaridae	*Pachypsylla venusta*	Olisa-AKH	pEVNFSPNWGGa	1087.4956	GAOP01010573
		*Aphalara polygoni*	Olisa-AKH	pEVNFSPNWGGa	1087.4956	GCWO01026629 (3);
		*Glycaspis brimblecombei*	Olisa-AKH	pEVNFSPNWGGa	1087.4956	GDFP01029145 (3);
	Triozidae	*Trioza urticae*	Olisa-AKH	pEVNFSPNWGGa	1087.4956	GCYX01025297 (3);
		*Bactericera trigonica*	Olisa-AKH	pEVNFSPNWGGa	1087.4956	GHUW01028007 (4);
		*Acanthocasuarina muellerianae*	Olisa-AKH	pEVNFSPNWGGa	1087.4956	GAYY02041518 (5);
	Liviidae	*Psyllopsis fraxinicola*	Olisa-AKH	pEVNFSPNWGGa	1087.4956	GCYW01003202 (3);
Sternorrhyncha, Aphidoidea	Aphididae	*Acyrthosiphon pisum*	Acypi-AKH	pEVNFTPTWGQa	1159.5531	(6)
		*Aphis gossypii*	Acypi-AKH	pEVNFTPTWGQa	1159.5531	(7)
		*Pseudoregma bambucicola*	Acypi-AKH	pEVNFTPTWGQa	1159.5531	(7)
		*Sitobion avenae*	Acypi-AKH	pEVNFTPTWGQa	1159.5531	(7)
		*Melanaphis sacchari*	Acypi-AKH	pEVNFTPTWGQa	1159.5531	XP_025194429
		*Myzus persicae*	Acypi-AKH	pEVNFTPTWGQa	1159.5531	XP_022166997
		*Sipha flava*	Acypi-AKH	pEVNFTPTWGQa	1159.5531	XP_025422856
		*Diuraphis noxia*	Acypi-AKH	pEVNFTPTWGQa	1159.5531	XP_015374547
		*Rhopalosiphum maidis*	Acypi-AKH	pEVNFTPTWGQa	1159.5531	XP_026812732
		*Cinara cedri*	Acypi-AKH	pEVNFTPTWGQa	1159.5531	VVC28311
		*Aphis glycines*	Acypi-AKH	pEVNFTPTWGQa	1159.5531	VYZN01000041.1
		*Aphis craccivora*	Acypi-AKH	pEVNFTPTWGQa	1159.5531	KAF0758447
		*Macrosiphum euphorbiae*	Acypi-AKH	pEVNFTPTWGQa	1159.5531	GAOM01002133
		*Schizaphis graminum*	Acypi-AKH	pEVNFTPTWGQa	1159.5531	GCZI01006870 (3);
		*Myzus cerasi*	Acypi-AKH	pEVNFTPTWGQa	1159.5531	Mc9227, AphidBase
		*Rhopalosiphum padi*	Acypi-AKH	pEVNFTPTWGQa	1159.5531	Rp1130, AphidBase
		*Eriosoma lanigerum*	Acypi-AKH	pEVNFTPTWGQa	1159.5531	(8)
		*Pentalonia nigronervosa*	Acypi-AKH	pEVNFTPTWGQa	1159.5531	g20381.p1, AphidBase
		*Uroleucon ambrosiae*	Acypi-AKH	pEVNFTPTWGQa	1159.5531	GCZC01009275 (3);
Sternorrhyncha, Aphidoidea	Adelgidae	*Dreyfusia ssp*	Acypi-AKH	pEVNFTPTWGQa	1159.5531	(7)
Sternorrhyncha, Coccoidea	Coccidae	*Ceroplastes cirripediformis*	**Novel 1**	pEVNFSPYWNQa	1264.5745	GCWZ01002139 (3);
		*Coccus* sp.	**Novel 1**	pEVNFSPYWNQa	1264.5745	GCWW01014892 (3);
	Diaspididae	*Unaspis euonymi*	**Novel 1**	pEVNFSPYWNQa	1264.5745	GCZJ01029341 (3);
		*Chrysomphalus aonidum*	**Novel 1**	pEVNFSPYWNQa	1264.5745	GCVU01019451 (3);
		*Aspidiotus rigidus*	**Novel 1**	pEVNFSPYWNQa	1264.5745	GHKI01018616 (9);
		*Aspidiotus destructor*	**Novel 1**	pEVNFSPYWNQa	1264.5745	GGKE01207341
		*Acutaspis umbonifera*	**Novel 1**	pEVNFSPYWNQa	1264.5745	GCXJ01023699 (3);
	Kerriidae	*Kerria lacca*	**Novel 2**	pEVNFTPYWNQa	1278.5902	GBDY01032366
	Pseudococcidae	*Planococcus citri*	**Novel 1**	pEVNFSPYWNQa	1264.5745	GAXF02026530 (5);
		*Trionymus caricis*	**Novel 1**	pEVNFSPYWNQa	1264.5745	GCYT01017461 (3);
	Aclerdidae	*Aclerda* sp.	**Novel 1**	pEVNFSPYWNQa	1264.5745	GCWU01017475 (3);
	Dactylopiidae	*Dactylopius confusus*	**Novel 1**	pEVNFSPYWNQa	1264.5745	GCWV01028377 (3);
Coleorrhyncha	Peloridiidae	*Xenophysella greensladeae*	Manto-CC	pEVNFSPGWa	916.4312	GAYI02027838 (5);
		*Hackeriella veitchi*	Manto-CC	pEVNFSPGWa	916.4312	GFEH01014874
		*Peloridium pomponorum*	Corpu-AKH	pELNFSPSWa	960.4574	GCZG01017621 (3);
		*Xenophyes metoponcus*	Manto-CC	pEVNFSPGWa	916.4312	GDEM01031637 (3);
Auchenorrhyncha, Fulgoroidea	Delphacidae	*Nilaparvata lugens*	Peram-CAH-I	pEVNFSPNWa	973.4526	(10, 11)
		*Laodelphax striatellus*	Peram-CAH-I	pEVNFSPNWa	973.4526	AXF48182.1 (12);
		*Sogatella furcifera*	Peram-CAH-I	pEVNFSPNWa	973.4526	GFWP01035542
		*Idiosystatus acutiusculus*	Peram-CAH-I	pEVNFSPNWa	973.4526	GCXZ01027840 (3);
Auchenorrhyncha, Fulgoroidea	Tropiduchidae	*Ladella* sp.	**Novel 3**	pEVNFTPGWGGa	1044.4898	GCXO01051348 (3);
Auchenorrhyncha, Fulgoroidea	Achilidae	*Catonia nava*	Anaim-AKH	pEVNFSPSWa	946.4417	GEHT01030223
	Dictyopharidae	*Chondrodire chilensis*	Anaim-AKH	pEVNFSPSWa	946.4417	GCYG01006298 (3);
		*Yucanda albida*	Anaim-AKH	pEVNFSPSWa	946.4417	GFEF01029762
		*Phylloscelis atra*	Anaim-AKH	pEVNFSPSWa	946.4417	GCYH01022542 (3);
		*Dictyophara europaea*	Anaim-AKH	pEVNFSPSWa	946.4417	GDEP01027356 (3);
	Derbidae	*Omolicna uhleri*	Anaim-AKH	pEVNFSPSWa	946.4417	GEIW01049815
	Cixiidae	*Melanoliarus placitus*	Anaim-AKH	pEVNFSPSWa	946.4417	GCZE01047419 (3);
		*Tachycixius pilosus*	Anaim-AKH	pEVNFSPSWa	946.4417	GDEX01003346 (3);
	Caliscelidae	*Bruchomorpha oculata*	Anaim-AKH	pEVNFSPSWa	946.4417	GCWF01037340 (3);
		*Caliscelis bonellii*	Anaim-AKH	pEVNFSPSWa	946.4417	GDFE01015571 (3);
	Flatidae	*Metcalfa pruinosa*	Anaim-AKH	pEVNFSPSWa	946.4417	GDFH01005752 (3);
		*Jamella australiae*	Anaim-AKH	pEVNFSPSWa	946.4417	GEJH01004521 (3);
	Tettigometridae	*Tettigometra bipunctata*	Pyrap-AKH	pELNFTPNWa	1001.4839	GFEJ01021282
	Fulgoridae	*Lycorma delicatula*	Corpu-AKH	pELNFSPSWa	960.4574	GFES01009373
		*Cyrpoptus belfragei*	Corpu-AKH	pELNFSPSWa	960.4574	GCWQ01018876 (3);
	Issidae	*Thionia simplex*	Anaim-AKH	pEVNFSPSWa	946.4417	GEIJ01039255
	Nogodinidae	*Lipocallia australensis*	Anaim-AKH	pEVNFSPSWa	946.4417	GEIY01015402
		*Bladina* sp.	Anaim-AKH	pEVNFSPSWa	946.4417	GFEA01003037
	Acanaloniidae	*Acanalonia conica*	Anaim-AKH	pEVNFSPSWa	946.4417	GCXC01059237 (3);
Auchenorrhyncha, Cicadoidea	Cicadidae	*Platypleura capensis*	Placa-HrTH*	pEVNFSPSWGNa	1117.5061	(13)
		*Munza trimeini*	Placa-HrTH*	pEVNFSPSWGNa	1117.5061	(13)
		*Cacama valavata*	Placa-HrTH*	pEVNFSPSWGNa	1117.5061	(14)
		*Diceroprocta semicincta*	Placa-HrTH*	pEVNFSPSWGNa	1117.5061	(14)
		*Magicicada* sp.	Placa-HrTH*	pEVNFSPSWGNa	1117.5061	(15)
		*Burbunga queenslandica*	Placa-HrTH	pEVNFSPSWGNa	1117.5061	GEJZ01015462
		*Okanagana villosa*	Placa-HrTH	pEVNFSPSWGNa	1117.5061	GAWQ02015184 (5);
		*Maoricicada tenuis*	Placa-HrTH **Novel 4**	pEVNFSPSWGNa pELTFSPSWGNa	1117.5061 1118.5265	GEJB01022642 GEJB01024815
		*Tamasa doddi*	Placa-HrTH	pEVNFSPSWGNa	1117.5061	GEIK01019861
		*Diceroprocta semicincta*	Placa-HrTH	pEVNFSPSWGNa	1117.5061	GGPH01043014
		*Kikihia scutellaris*	**Novel 4**	pELTFSPSWGNa	1118.5265	GEJF01000577
	Tettigarctidae	*Tettigarcta crinita*	Placa-HrTH	pEVNFSPSWGNa	1117.5061	GEHU01015427
Auchenorrhyncha, Cercopoidea	Cercopidae	*Locris arithmetica*	Peram-CAH-I Pyrap-AKH	pEVNFSPNWa pELNFTPNWa	973.4526 1001.4839	(16)
		*Cercopis vulnerata*	Peram-CAH-I Emppe-AKH	pEVNFSPNWa pEVNFTPNWa	973.4526 987.4683	(5, 17) GAUN02023904 (5);
		*Prosapia bicincta*	Psein-AKH Peram-CAH-I	pEVNFTPGWa pEVNFSPNWa	930.4468 973.4526	GCYJ01017491 (3); GCYJ01021165 (3);
Auchenorrhyncha, Cercopoidea	Aphrophoridae	*Ptyelus flavescens*	Peram-CAH-I Ptyfl-AKH-I Ptyfl-AKH-II	pEVNFSPNWa pEINFSTGWGQa pEINFSTAWGQa	973.4526 1119.5218 1133.5374	(18)
		*Aphrophora alni*	Emppe-AKH Peram-CAH-I	pEVNFTPNWa pEVNFSPNWa	987.4683 973.4526	(3, 17) GDEN01003359 (3);
		*Philaenus spumarius*	Peram-CAH-I	pEVNFSPNWa	973.4526	GCZA01031114 (3);
Auchenorrhyncha,Membracoidea	Cicadellidae	*Cicadella viridis*	Anaim-AKH	pEVNFSPSWa	946.4417	(17)
		*Graphocephala fennahi*	Anaim-AKH	pEVNFSPSWa	946.4417	(17)
		*Graminella nigrifrons*	Anaim-AKH	pEVNFSPSWa	946.4417	GAQX01004248 (19);
		*Vidanoana flavomaculata*	Anaim-AKH	pEVNFSPSWa	946.4417	GCZD01028657 (3);
		*Dalbulus maidis*	Anaim-AKH	pEVNFSPSWa	946.4417	GHCC01023424
		*Agudus* sp.	Anaim-AKH	pEVNFSPSWa	946.4417	GFDX01034468
		*Cuerna arida*	Anaim-AKH	pEVNFSPSWa	946.4417	GECZ01031106
		*Amrasca biguttula biguttula*	Anaim-AKH	pEVNFSPSWa	946.4417	GGAG01048354
		*Agallia constricta*	Anaim-AKH	pEVNFSPSWa	946.4417	GCWT01020143 (3);
		*Ulopa reticulata*	Anaim-AKH	pEVNFSPSWa	946.4417	GDEO01044873 (3);
		*Empoasca fabae*	Anaim-AKH	pEVNFSPSWa	946.4417	GCVV01009899 (3);
		*Nionia palmeri*	Anaim-AKH	pEVNFSPSWa	946.4417	GEKN01042692
		*Penthimia* sp.	Anaim-AKH	pEVNFSPSWa	946.4417	GFFF01070643
		*Euacanthella palustris*	Anaim-AKH	pEVNFSPSWa	946.4417	GEJL01012714
		*Hespenedra chilensis*	Libau-AKH **Novel 5**	pEVNFTPSWa pEVNFTPYWa	960.4574 1036.4887	GCXG01036502 (3); GCXG01005578 (3);
		*Macropsis decisa*	Anaim-AKH	pEVNFSPSWa	946.4417	GEJE01013300
		*Aphrodes bicincta*	Anaim-AKH	pEVNFSPSWa	946.4417	GFDZ01090351
		*Ponana quadralaba*	Anaim-AKH	pEVNFSPSWa	946.4417	GCZF01074577 (3);
		*Xestocephalus desertorum*	Anaim-AKH	pEVNFSPSWa	946.4417	GELC01062483
		*Neocoelidia tumidifrons*	Anaim-AKH	pEVNFSPSWa	946.4417	GEJC01018468
		*Tituria crinita*	Anaim-AKH	pEVNFSPSWa	946.4417	GFEI01042457
		*Idiocerus rotundens*	Peram-CAH-I	pEVNFSPNWa	973.4526	GEJG01013906
		*Penestragania robusta*	Anaim-AKH	pEVNFSPSWa	946.4417	GFFC01022604
		*Tinobregmus viridescens*	Anaim-AKH	pEVNFSPSWa	946.4417	GEIL01023531
		*Homalodisca vitripennis*	Anaim-AKH	pEVNFSPSWa	946.4417	SRR14298121 (20);
Auchenorrhyncha,Membracoidea	Membracidae	*Procyrta* sp.	Anaim-AKH	pEVNFSPSWa	946.4417	GFFD01030962.1
		*Centrotus cornutus*	Anaim-AKH	pEVNFSPSWa	946.4417	GDFF01029196 (3);
		*Heteronotus* sp.	Anaim-AKH	pEVNFSPSWa	946.4417	GEJQ01007420
		*Enchenopa latipes*	Libau-AKH	pEVNFTPSWa	960.4574	GCWI01029089 (3);
		*Holdgatiella chepuensis*	Peram-CAH-I	pEVNFSPNWa	973.4526	GCXW01013215 (3);
		*Chelyoidea sp*	Anaim-AKH	pEVNFSPSWa	946.4417	GFEB01065829
		*Nessorhinus gibberulus*	Anaim-AKH	pEVNFSPSWa	946.4417	GCYF01016217 (3);
		*Microcentrus caryae*	Anaim-AKH	pEVNFSPSWa	946.4417	GEIX01026642
		*Lycoderes burmeisteri*	Anaim-AKH	pEVNFSPSWa	946.4417	GFER01033440
		*Cyphonia clavata*	Anaim-AKH	pEVNFSPSWa	946.4417	GFEC01041949
		*Stictocephala bisonia*	Anaim-AKH	pEVNFSPSWa	946.4417	GCZK01023978 (3);
Auchenorrhyncha,Membracoidea	Melizoderidae	*Llanquihuea pilosa*	Peram-CAH-I	pEVNFSPNWa	973.4526	GCWX01015088 (3);
Auchenorrhyncha,Membracoidea	Myerslopiidae	*Mapuchea* sp.	Anaim-AKH	pEVNFSPSWa	946.4417	GCXN01065091 (3);
Auchenorrhyncha,Membracoidea	Aetalionidae	*Lophyraspis* sp.	Peram-CAH-I	pEVNFSPNWa	973.4526	GFEQ01009937
Heteroptera, Gerromorpha, Gerroidea	Gerridae	*Gerris lacustris*	Grybi-AKH	pEVNFSTGWa	920.4261	(21)
		*Limnoporus canaliculatus*	Grybi-AKH	pEVNFSTGWa	920.4261	GCYA01035739 (3);
		*Aquarius paludum*	Grybi-AKH	pEVNFSTGWa	920.4261	GDFO01022466 (3);
		*Gerris buenoi*	Grybi-AKH	pEVNFSTGWa	920.4261	JHBY02063394
Heteroptera, Nepomorpha, Corixoidea	Corixidae	*Corixa punctata*	Corpu-AKH	pELNFSPSWa	960.4574	(22)
		*Trichocorixa calva*	Corpu-AKH	pELNFSPSWa	960.4574	GCYZ01011904 (3);
Heteroptera, Nepomorpha, Nepoidea	Nepidae	*Nepa cinerea*	Nepci-AKH	pELNFSSGWa	920.4261	(23)
		*Ranatra linearis*	Grybi-AKH	pELNFSPSWa	920.4261	(23)
		*Laccotrephes fabricii*	Peram-CAH-I	pEVNFSPNWa	973.4526	(24)
	Belostomatidae	*Lethocerus indicus*	Letin-AKH	pEVNFSPYWa	1022.4730	(23)
		*Hydrocyrius columbiae*	Letin-AKH Anaim-AKH	pEVNFSPYWa pEVNFSPSWa	1022.4730 946.4417	(24)
		*Appasus grassei*	Anaim-AKH	pEVNFSPSWa	946.4417	(24)
Heteroptera, Nepomorpha, Ochteroidea	Gelastocoridae	*Gelastocoris oculatus*	Anaim-AKH	pEVNFSPSWa	946.4417	GCXI01021875 (3);
Heteroptera, Nepomorpha, Naucoroidea	Naucoridae	*Ilyocoris cimicoides*	Anaim-AKH	pEVNFSPSWa	946.4417	(22)
		*Laccocoris spurcus*	Lacsp-AKH	pEVNFSPSWGGa	1060.4847	(25)
	Aphelocheiridae	*Aphelocheirus aestivalis*	Anaim-AKH	pEVNFSPSWa	946.4417	(25)
						
Heteroptera, Nepomorpha, Notonectoidea	Notonectidae	*Notonecta glauca*	Anaim-AKH	pEVNFSPSWa	946.4417	(26)
		*Notonecta obliqua*	Anaim-AKH	pEVNFSPSWa	946.4417	(27)
		*Notonecta reuteri*	Anaim-AKH	pEVNFSPSWa	946.4417	(27)
		*Buenoa margaritacea*	Anaim-AKH	pEVNFSPSWa	946.4417	GCXE01046849 (3);
Heteroptera, Leptopodomorpha, Saldoidea	Saldidae	*Saldula saltatoria*	Tippa-CC-II	pELTFSPSWa	947.4621	GDER01017547 (3);
Heteroptera, Cimicomorpha, Reduvioidea	Reduviidae	*Rhodnius prolixus*	Rhopr-AKH	pELTFSTDWa	979.4520	(28, 29)
		*Panstrongylus megistus*	Rhopr-AKH	pELTFSTDWa	979.4520	(29)
		*Triatoma infestans*	Triin-AKH	pELTFTPNWGa	1045.5102	(29)
		*Dipetalogaster maxima*	Dipma-AKH	pELTFSDGWGNa	1106.4901	(29)
Heteroptea, Cimicomorpha, Cimicoidea	Cimicidae	*Cimex lectularius*	Cimle-AKH	pEITFSTGWa	921.4465	(30)
Heteroptea, Cimicomorpha, Cimicoidea	Anthocoridae	*Orius insidiosus*	Dircl-AKH-II	pELTFSTGWa	921.4465	JACGAQ010000095
		*Orius laevigatus*	Dircl-AKH-II	pELTFSTGWa	921.4465	JAGWEN010000368 (31);
Heteroptera, Cimicomorpha, Miroidea	Miridae	*Lygus hesperus*	**Novel 6** **Novel 7**	pELTYSPNWa pELTYSPGWa	990.4680 933.4465	QQW38901 (32); QQW38902 (32);
		*Apolygus lucorum* (other name: *Lygus lucorum*)	Glomo-AKH Dircl-AKH-II	pELTFSPGWa pELTFSTGWa	917.4516 921.4465	KAF6197472 (33); WIXP02000008 (33);
		*Adelphocoris suturalis*	**Novel 8**	pELTYSTNWa	994.4629	GGBU01033998
		*Adelphocoris lineolatus*	**Novel 8**	pELTYSTNWa	994.4629	GGBQ01001787
		*Notostira elongata*	Diptera novel	pELTFSPNWa	974.4730	GASV02006982 (5);
		*Tupiocoris notatus*	Peram-CAH-II	pELTFTPNWa	988.4887	GFBA01048599
		*Lopidea amorphae*	**Novel 8**	pELTYSTNWa	994.4629	GCXF01011160 (3);
		*Reuteroscopus ornatus*	**Novel 9**	pELTYSPNWGa	1047.4894	GCYK01002075 (3);
		*Nesidiocoris tenuis*	Peram-CAH-II	pELTFTPNWa	988.4887	CADCXU010005909
		*Cyrtorhinus lividipennis*	**Novel 6**	pELTYSPNWa	990.4680	JADDOF010000010
Heteroptera, Pentatomomorpha, Pentatomoidea	Pentatomidae	*Nezara viridula*	Panbo-RPCH Nezvi-AKH**^#^**	pELNFSPGWa pELNFSPGWa	930.4468 946.4417	(34) (35)
		*Coenomorpha ssp*	Panbo-RPCH	pELNFSPGWa	930.4468	(27)
		*Eurydema oleracea*	Panbo-RPCH	pELNFSPGWa	930.4468	(27)
		*Eurydema ornata*	Panbo-RPCH	pELNFSPGWa	930.4468	(27)
		*Rhaphigaster nebulosa*	Panbo-RPCH	pELNFSPGWa	930.4468	(27)
		*Graphosoma lineatum*	Panbo-RPCH	pELNFSPGWa	930.4468	(27)
		*Acrosternum hilare*	Panbo-RPCH	pELNFSPGWa	930.4468	(36)
		*Euschistus servus*	Panbo-RPCH	pELNFSPGWa	930.4468	(36)
		*Banasa dimiata*	Panbo-RPCH	pELNFSPGWa	930.4468	(36)
		*Plautia stali*	Panbo-RPCH	pELNFSPGWa	930.4468	BAV78787.1
		*Halyomorpha halys*	Panbo-RPCH	pELNFSPGWa	930.4468	JMPT03100225
		*Aelia acuminata*	Panbo-RPCH	pELNFSPGWa	930.4468	ERR6054995
	Acanthosomatidae	*Acanthosoma haemorrhoidale*	Schgr-AKH-II	pELNFSTGWa	934.4417	(27)
	Tessaratomidae	*Encosternum delegorguei*	Panbo-RPCH Schgr-AKH-II	pELNFSPGWa pELNFSTGWa	930.4468 934.4417	(16)
		*Tessaratoma papillosa*	Panbo-RPCH	pELNFSPGWa	930.4468	SRX12310643
Heteroptera, Pentatomomorpha, Pyrrhocoroidea	Pyrrhocoridae	*Pyrrhocoris apterus*	Pyrap-AKH Peram-CAH-II	pELNFTPNWa pELTFTPNWa	1001.4839 988.4887	(27)
		*Cenaeus carnifex*	Pyrap-AKH	pELNFTPNWa	1001.4839	(27)
		*Dysdercus intermedius*	Pyrap-AKH	pELNFTPNWa	1001.4839	(27)
		*Dysdercus cingulatus*	Pyrap-AKH	pELNFTPNWa	1001.4839	(27)
Heteroptera, Pentatomomorpha,Coreoidea	Coreidae	*Holopterna alata*	Schgr-AKH-II Schgr-AKH-II**^Ŧ^**	pELNFSTGWa pELNFSTGWa	934.4417 1014.3986	(37) (38)
		*Anoplocnemis ssp*	Schgr-AKH-II	pELNFSTGWa	934.4417	(27)
		*Coreus marginatus*	Schgr-AKH-II	pELNFSTGWa	934.4417	(27)
Heteroptera, Pentatomomorpha,Coreoidea	Stenocephalidae	*Dicranocephalus agilis*	Schgr-AKH-II	pELNFSTGWa	934.4417	(27)
Heteroptera, Pentatomomorpha,Lygaeoidea	Lygaeidae	*Oncopeltus fasciatus*	Tenmo-HrTH	pELNFSPNWa	987.4683	(39)
		*Lygaeus kalmii*	Tenmo-HrTH	pELNFSPNWa	987.4683	Gäde G, Simek P, Marco HG unpublished

Genbank (NCBI) and AphidBase Accession numbers supplied. AphidBase genomes were accessed from the BIPAA (Bioinformatics Platform for Agroecosystem Arthropods) website: https://bipaa.genouest.org/is/

The occurrence of 9 novel AKH sequences is indicated in bold text as Novel.

*Two peptides with an identical sequence and mass, but with different retention times on LC.

**^#^** Represents a hydroxyproline form of Panbo-RPCH.

**^Ŧ^** Represents a sulphated form of Schgr-AKH-II.

## 2 The Main Players

### 2.1 The Order Hemiptera and the Chief Pests Therein

The order Hemiptera is quite often referred to as “true bugs”, although others reserve this term for the suborder Heteroptera (see **Figure 1**). We all probably know of hemipterans called aphids (Superfamily Aphidoidea), whiteflies (Superfamily Aleyrodoidea), scale insects (Superfamily Coccoidea), cicadas (Superfamily Cicadoidea), water striders (Superfamily Gerroidea), back swimmers (Superfamily Notonectoidea), bed bugs (Superfamily Cimicoidea), kissing bugs (Superfamily Reduvioidea) and stink bugs (Superfamily Pentatomoidea), but not many laypersons or non-specialist hemipteran taxonomists have any association with hemipteran insects when they read of psyllids (Superfamily Psylloidea), moss bugs (Suborder Coleorrhyncha), planthoppers (Superfamily Fulgoroidea), spittlebugs (Superfamily Cercopoidea), treehoppers (Superfamily Membracoidea), aquatic stick insects (Superfamily Nepoidea), water boatman (Superfamily Corixoidea), damsel bugs (Superfamily Naboidea), twig wilters (Superfamily Coreoidea) or seed bugs (Superfamily Lygaeoidea). This impressive list of “bugs” is indicative of the species-richness of Hemiptera. Hence, it does not surprise that Hemiptera comprises the largest order of the exopterygote (hemimetabolous) insects with more than 80 000 described species (40). Together with the other two hemipteroid orders, Psocodea (bark and parasitic lice) and Thysanoptera (thrips), the number of described species rises to an impressive over 120 000 which relates to more than 10% of known insects (3). The importance of a robust phylogenetic treatment of Hemiptera to provide a framework for interpreting and understanding morphological transitions, behavioral, metabolic and environmental processes, for example, in an evolutionary context, has interested scientists for a long time; a few recent examples are (5, 41–44). The latest and most comprehensive phylogenomic study takes a dataset of 2 395 protein-coding single-copy genes from more than 160 hemipteroid and outgroup (33 species) taxa into account (3).

**Figure 1** is a somewhat simplified and condensed version of the phylogenetic tree of Hemiptera as proposed by (3). It shows the major suborders and superfamilies and serves as orientation for the reader. Note that we have labelled only those branches in which AKH sequences could be retrieved from, e.g. Infraorders Dipsocoromorpha and Enicocephalomorpha are not shown in **Figure 1**. In this phylogeny, the orders Thysanoptera (thrips) and Hemiptera are monophyletic and sister groups. The suborder Sternorrhyncha is the sister clade to all other hemipterans, and within the Sternorrhyncha the superfamily Aleyrodoidea (whiteflies) is the sister group to the other three superfamilies, viz. Psylloidea (psyllids), Aphidoidea (aphids) and Coccoidea (scale insects). All these superfamilies contain pest insects that damage agricultural crop plants and/or horticultural ornamental plants. Quite often the damage is not done by depriving the plant of its phloem or xylem sap but by the transmission of plant viruses (45).

An infamous example of a pest insect from the Aleyrodoidea is the sweet potato or silverleaf whitefly, *Bemisia tabaci*, which is a species complex and does not only damage the various food plants but is also a vector for transmitting plant viruses (46). Furthermore, the whitefly is quite resistant to most insecticides in use. Therefore, the elucidation of its genome has given hope to new strategies of combatting this species, including methodologies involving neuropeptides (47, 48).

The same is true for one of the worst pests of citrus fruits worldwide belonging to the Psylloidea, the Asian citrus psyllid *Diaphorina citri*, which is a vector of a phloem-inhabiting bacterium responsible for the “citrus greening disease” for which no recipe to kill exists, thus efforts are to possibly construct and use neuropeptide-based insecticides against the vector which is already becoming resistant against common chemical-based insecticides (2, 49, 50).

The superfamily Aphidoidea accommodates many thousands of aphid species that can reproduce by parthenogenesis or sexually, and many aphid species are serious crop pests and vectors for a large number of viruses; well-known members of the aphids are the green peach aphid, *Myzus persicae*, the pea aphid, *Acyrthosiphon pisum*, and the cotton and melon aphid, *Aphis gossypii*. The whole genome is now sequenced from several aphid species after the very first hemipteran genome was completed from *A. pisum* in 2010 (51–55). This means, thus, that gene information on the neuropeptide precursors and G protein-coupled receptors of several aphid species may be obtained from the genomes (see **Table 1** for database accession numbers or published references of genomes and transcriptomes, and chemically elucidated sequences).

Members of the Coccoidea, also called scale insects because the whole body is covered by a scale, are quite strange insects, being mostly sessile, reproduce often parthenogenetically and, as herbivores, are quite damaging to certain plants (especially citrus fruits by *Icerya purchasi*) but are mostly inaccessible to insecticides due to their scale and waxy surfaces. The other side of the coin, however, are some beneficial traits of Coccoidea: certain species are used as biocontrol agents in South Africa especially against the invasive *Opuntia* cactus, the prickly pear (56), and other species are sought after for their red dyes and shellac, a resin that was formerly used to produce long play records but is still used in various manufacturing processes.

Not much is known of the moss feeding moss bugs (Suborder Coleorrhyncha), of which only a single family apparently is extant. Nonetheless, Coleorrhyncha is the sister group to the monophyletic suborder Auchenorrhyncha which is divided into two infraorders: Fulgomorpha (with the superfamily Fulgoroidea = planthoppers) and Cicadomorpha (with three superfamilies: Cicadoidea = cicadas, Cercopoidea = spittlebugs and Membracoidea = leaf- and treehoppers), **Figure 1** (3).

There are more than 12 000 known species of planthoppers, that feed off plant sap as adults and nymphs, thereby producing honeydew which is often related with fungal growth. Mostly, the Fulgoroidea do not have a declared pest status although some are vectors for plant diseases which are transmitted during feeding as outlined below. The superfamily encompasses a number of families, of which we will introduce here only the Delphacidae because it contains the brown planthopper, *Nilaparvata lugens*, a dimorphic (macropterous and brachyopterous forms exist) most harmful pest insect for rice species (*Oryza sativa*) where the damage is done through extensive feeding and viral transmission (57, 58). Two other delphacid planthoppers, *Sogatella furcifera* and *Laodelphx striatellus*, are responsible for transmission of rice viruses. Genomic information is now available from all three species (10, 12, 59) and the analyses of neuropeptides and their receptor sequences were carried out with the transcriptome of *N. parvata* (11). This paves the way for experimentation on the cellular and molecular levels to provide insights into regulation of physiological/biological cycles in these tiny insects, and possible ways to disrupt these cycles.

Whereas adult cicadas (Superfamily Cicadoidea) are largely viewed as non-destructive insects without pest status (at least if you are not supersensitive to their shrill calling songs), their nymphal stages –which may develop for up to 17 years, as in the genus *Magicicada* – are quite voracious feeders on root systems of certain plants and can be very destructive, especially with occasional outbreaks of *Magicicada*. The superfamily Cercopoidea has many species that are well known for their powerful jumping ability; this has been studied in species of the froghopper *Cercopis vulnerata* and the spittle bugs *Philaenus spumariu*s and *Aphrophora alnus* (60–63). These species are all known for severely damaging various grass species (*C. vulnerata*), clover and alfalfa (*P. spumarius*) and mostly willow and olive trees (*A. alni*) (64, 65). The superfamily Membracoidea are plant suckers as well and contains mainly the two families Membracidae, the treehoppers, and Cicadellidae, the leafhoppers. Both families have, in general, species that are more or less innocuous to humans and, if at all, may be considered minor pest insects, such as the three-cornered alfalfa hopper *Spissistilus festinus* (Family Membracidae) (66). Although the green leafhopper, *Cicadella viridis* (Family Cicadellidae), is polyphagous and feeds on a number of different herbaceous plants, it does not produce major damages. Similarly, the rhododendron leafhopper, *Graphocephala fennahi*, a native of the USA which was introduced to Europe as late as in the 1970s, is sometimes thought of spreading the rhododendron fungus that affects the popular garden shrub, but other research could not establish evidence to substantiate such a claim (67).

The sister group to the Auchenorrhyncha/Coleorrhyncha is the species-rich (about 40 000 species) suborder Heteroptera with 7 recognized infraorders of which at least 4 were monophyletic in the study of (3). Heteroptera contain mostly semi aquatic (Infraorder Gerromorpha and Leptopodomorpha), aquatic (Infraorder Nepomorpha) and terrestrial clades (Infraorders Pentatomorpha and Cimicomorpha, both of which are known to harbour true pest insect species) plus the two most basal heteropteran infraorders, the not well-researched and species-poor (only 70 known species) Enicocephalomorpha and the minute Dipsocoromorpha with only about 350 extant species. The Gerromorpha is sister group to the latter infraorders (3), and one of its superfamilies is the Gerroidea of which about 2000 species exist including the family of the predatory Gerridae, the water striders, known for their ability to “walk on water” because of distinct morphological and biomechanical adaptations (68). Quite recently the genome of the species *Gerris buenoi* was unravelled (69). Although the Nepomorpha also contain only about 2000 species, there are some well-known members even recognized by the general public, such as the water boatmen (Superfamily Corixoidea, Family Corixidae), the water scorpions (Superfamily Nepoidea, Family Nepidae) and the backswimmers (Superfamily Notonectoidea, Family Notonectidae) (70). The Nepomorpha species are aquatic, mostly predatory insects that swim well (some even upside down as the backswimmers) but are also capable of flight. Some have peculiar ways of breathing air while submerged: Family Notonectidae, for example, dive underwater with an air bubble positioned to contact one or more pairs of spiracles; this air bubble functions as a physical gill and as an oxygen store - during diving, oxygen diffuses from the air bubble into the tracheal system and to the cells, while carbon dioxide is released from the body and diffuses into the surrounding water. As the volume of the bubble shrinks, oxygen diffuses into the air bubble from the surrounding water. Because of a very dense body coverage with microtrichia, the air bubble is held in place and water cannot flood into the spiracles (71). The water bug *Aphelocheirus* (Superfamily Naucoroidea, Family Aphelocheiridae) can stay permanently under water breathing atmospheric air from a so-called plastron which is an incompressible physical gill composed of a thin layer of hydrophobic cuticular hairs or scales with a bent tip that traps a film of oxygen around the body surface; the tracheae can access the air in the plastron while the insect is underwater (72). About half of the heteropteran species belong to the infraorder Cimicomorpha (about 20 000 extant species) which *inter alia* harbour such important and well-known superfamilies as the Reduvioidea (assassin bugs), Cimicoidea (bed bugs) and Miroidea (plant bugs). Bugs of the family Reduviidae are infamous for feeding on vertebrate blood, and the kissing bugs such as *Rhodnius prolixus* and *Triatoma infestans* (Subfamily Triatominae) are vectors of Chagas disease caused by infection with the protozoan parasite *Trypanosoma cruzi*. This disease affects about 7 million people in the Americas. The genome of *R. prolixus* is completely unravelled (73) and neuropeptide genes are identified (28). Individuals of the family Cimicidae are parasitic and also hemotaphagous, feeding mainly on the blood of bats, birds and humans; its best-known member is the common bedbug, *Cimex lectularius*, which causes an immune response in the human host and discomfort but seldom transmits viruses (74). Genomic information, as well as the neuropeptidome of the bedbug is available since the last few years (30, 75, 76). The largest heteropteran family of plant bugs, the Miridae (Superfamily Miroidea), is noteworthy as it contains very serious agricultural pests, such as the tarnish plant bug *Lygus hesperus*, damaging to crops such as alfalfa and cotton (77). The species *Apolygus lucorum* is more or less omnivorous and damages many different plant species; its genome has been elucidated (33). On the other hand, there are Miridae species that are predatory, thus not phytophagous, and beneficial as biological control agents, as is the case with *Nesidiocoris tenuis*, also called the tomato bug, which is mainly used in tomato greenhouses to devour whiteflies, and whose genome was recently published (78). Or the mirid *Cyrtorhinus lividipennis* which is a predator of plant- and leafhoppers in rice fields and has its genome clarified (79). The last mirid of whom the genome is apparently known is *Pachypeltis micranthus*, which is thought to be used as a control agent against the invasive climbing hemp vine (*Mikania micrantha*) (80).

The sister group of the Cimicomorpha is the Pentatomorpha with its five superfamilies: the stink bugs (Pentatomoidea), red bugs (Pyrrhocoroidea), leaf-footed bugs (Coreoidea) and, lastly, the seed bugs (Lygaeoidea) (3). There are three families of interest in the Pentatomoidea all of which are characterized by chemical defenses and the shield-like shape of the body: the shield and giant shield bugs (Acanthosomatidae and Tessaratomidae) with only a few species (about 200) and the stink bugs (Pentatomidae) with more than 4700 species. Whereas the Acanthosomatidae are no real agricultural crop pest, Tessaratomidae and especially Pentatomidae cause enormous economic damage to agriculture, with the Tessaratomidae having an additional economic value as proteinaceous food for humans (81). Genomics and in some cases transcriptomics and neuropeptidomics has been applied to the pentatomid members *Aelia acuminata*, *Halyomorpha halys* and *Nezara viridula* (82–84). The family Pyrrhocoridae is well known for its red bug and especially the Linden bug *Pyrrhocoris apterus* that feeds on Linden and other seeds, while the cotton bug (genus *Dysdercus*) sucks on cotton seeds. The largest family of the superfamily Coreoidea is the Coreidae with almost 2000 robust looking species that also have scent glands releasing a chemical defense substance; these insects (e.g. *Holopterna alata*) feed on sap from the youngest shoots of plants causing wilting damage, which has led to their common name, twig wilters. The last superfamily of seed and sap feeding seed bugs, the Lygaeoidea, is quite large and contains many families of which the Lygaeidae with its most prominent member, the large milkweed bug *Oncopeltus fasciatus*, is best known. Research on this species centers on its diet, milkweed seed, and the sequestration of the toxins therein, which it uses as defense mechanism; its unpalatability is advertised with aposematic colouration (85). There are northern and southern populations of *O. fasciatus* in the USA that differ in their migration behaviour: the northern bugs undertake long distance flights (86). A genome project on this species has also been concluded (87).

### 2.2 The Adipokinetic Hormone Peptide Family

The most apt way to introduce this topic is to shortly talk about flight metabolism, for insect flight is indirectly regulated by the action of adipokinetic hormones. Flight in its sustained muscle-powered form is known to have occurred by convergent evolution in three extant animal lineages: the insects, birds and bats with the insects being the first in such an evolutionary event that was probably meant to evade predators and had occurred during a dramatic rise in the atmospheric oxygen concentration to 35% during the late Devonian to late Carboniferous period (88). One cannot, of course, associate hemipteran species as being especially fast flyers like the lepidopteran skippers (Hesperioidea) and sphinx moths (Bombycoidea) (see 89) or as prize-winning long-distance migrants such as certain locusts (90) and the Monarch butterfly (91). There are, however, some remarkable flight performances known from hemipteran species. The rice pest planthoppers of the family Delphacidae (Auchenorrhyncha, Fulgoroidea) cover about 1000 km from the Asian mainland to Japan, with the prevailing winds (see 92). Species of the basal Sternorrhyncha can also perform “long-distance” flights, albeit only for maximally 2 to 5 km, as deduced from experiments on specially constructed flight mills; for such tiny insects as the Asian citrus psyllid, *D. citri* (Superfamily Psylloidea), or the soybean aphid, *A. glycines* (Superfamily Aphidoidea), these are, indeed, long distances (93, 94). A caveat: the pros and cons of measuring tethered flight on a flight mill and the type of parameters that can be realistically deduced from such experiments, e.g. flight distance and speed, is reviewed by (95). Most superfamilies of the highest hemipteran suborder (Heteroptera) also have species that fly well over a distance for dispersal reasons. For example, aquatic bugs of the superfamilies Corixoidea and Nepoidea (96, 97), or tarnished plant bugs of the genus *Lygus* (Superfamily Miroidea) (98, 99), the stink bugs *H. halys* and *N. viridula* of the superfamily Pentatomoidea (100–103) and *O. fasciatus* of the superfamily Lygaeoidea (104). These are only a few examples. There are, however, also some species that we will cover later that are wingless and, hence, cannot fly at all like the bedbug *C. lectularius* (Superfamily Cimicoidea) and the Linden bug *P. apterus* (Superfamily Pyrrhocoroidea) (105, 106).

Regardless of the flight distance, insect flight is always using oxidative metabolism; the insect flight muscles are, thus, the most metabolically active tissues known in nature; for example, energetic output, measured as oxygen consumption during flight, can be elevated 100-fold above resting level (see reviews by 107, 108). To power this output of flight muscles, insects store metabolic fuel in the fat body, mainly as triacylglycerides (TAG) and/or as glycogen, and mobilize these stores hormonally by peptides belonging to the adipokinetic hormone family (see 108). The first insect adipokinetic hormone, today called Locmi-AKH-I, was chemically fully elucidated from the neurosecretory corpora cardiaca (CC) glands of the migratory (*Locusta migratoria*) and desert (*Schistocerca gregaria*) locusts (109). The first AKH gene was cloned from the tobacco hornworm moth, *Manduca sexta* (110). Generally, the AKH gene codes mRNA that is translated into a pre-propeptide with the following properties: a signal peptide followed immediately by the respective AKH, a glycine which functions as amidation site, a dibasic processing site and a C-terminal peptide of variable length with no known function. Cleavage and post-translational modification follows and shapes the structure of a mature AKH with the following characteristic features: the chain length is between 8 and 10 amino acids, the termini are blocked (a pyroglutamate at the N-terminus and a carboxyamide at the C-terminus), position two is either taken up by an aliphatic leucine, isoleucine or valine residue or by an aromatic such as phenylalanine or tyrosine, at position 3 one finds either asparagine or threonine, at position 4 phenylalanine or tyrosine, at position 5 threonine or serine, the signature amino acid tryptophan is always at position 8 and at position 9 is a glycine, while positions 6, 7 and 10 may be any of a wide variety of amino acids (see 21, 111). After release from the CC AKHs travel in the hemolymph to the target organ, notably the fat body, and exert their activity by binding to a G protein-coupled receptor, the first of which were identified genomically in the vinegar fly *Drosophila melanogaster* and the silk moth *Bombyx mori* (112, 113). Signal transduction has been studied for a number of species (reviewed by 114); in the case of regulation of lipid metabolism by AKHs, triacylglycerol (TAG) lipase activity results in the production and release of diacylglycerols (DAGs) into the hemolymph (see 115–118). The transport of this hydrophobic molecule to the flight muscles is complex: high density lipophorin associates with apolipophorin III and is loaded with DAGs, this creates a less dense protein particle, a low density lipophorin (see 115); in some cases, like in female *R. prolixus* undergoing oogenesis, lipophorin may not be converted into a low-density form (119). A membrane-bound lipoprotein hydrolyses the DAG at the flight muscles and the resulting fatty acids are oxidized to power the contraction for flight.

How does one identify a member of the AKH family in an insect species? In our hands and opinion, the easiest and most successful way is first to establish presence and approximate abundance of AKH material by using a functional assay to show that an extract of the CC (site of AKH synthesis and storage) can mobilize metabolites in a heterospecific insect, such as locusts, where AKHs have a demonstrated lipid-mobilizing (adipokinetic) effect, or cockroaches that respond to AKHs with an elevated carbohydrate concentration in the hemolymph (a hypertrehalosemic/hyperglycemic effect). Such heterologous bioassays are robust and proven (see 120). Socha et al. (121) are in favour of a conspecific biological test using the insect under study. Although this makes absolute sense from a physiological view, it may not be without complications since many insect species are not amenable to the necessary handling steps in such a biological assay, e.g. some insects are simply too small or too fragile for injection and sampling, while many others have fast clotting hemolymph when in contact with oxygen, thus making it impossible to measure the metabolite concentrations.

Once AKH presence is confirmed in an insect species, one may want to first isolate the AKH material from the CC extract *via* chromatographic means and then proceed with sequencing, or one may target and sequence the AKH without any prepurification/peptide isolation steps. Sequence information was relatively tedious to obtain a few decades ago when it was imperative to first isolate and purify the AKH *via* HPLC (high-pressure liquid chromatography) and then deblock the N-terminal pyroglutamate enzymatically before further analyses with an amino acid sequencer. Nowadays sequencing is easier by mass spectrometry and *via* data mining of publicly available bioinformatic information (genomic and transcriptomic databases). However, the “modern” methods of sequencing require additional corroborating steps since there are inherent shortcomings, such as assigning an identity to isobaric amino acids, or not being able to predict all post-translational modifications from a preprohormone translated mRNA sequence.

## 3 Structural and Functional Survey of Hemipteran AKHs

### 3.1 AKH-Regulated Metabolism in Hemiptera: The Beginnings

The high resolution power of the electron microscope was able to show abundant lipid droplets in muscles of the giant water bug (genus *Lethocerus*, Family Belostomatidae) (122). It took 14 years for a follow up in the triatomine bugs *R. prolixus* and *T. infestans* (Family Reduviidae): Ward et al. (123) studied the flight of the kissing bugs on specially constructed flight mills and found large amounts of lipids (as TAGs) stored in the flight muscles; upon flights of a 1 h duration, the concentration of the TAGs decreased significantly in the flight muscles with a concomitant increase of the level of DAGs in the hemolymph. On the other hand, concentrations of glycogen in the flight muscles and trehalose in the hemolymph remained low. Thus, here we have the first study in Hemiptera clearly demonstrating the exceptional role of lipid oxidation to fuel flight muscle contraction. What was missing at this early stage was to look at the regulation of the breakdown of TAGs and find an involvement of an AKH which could then have been identified. It would take almost another 30 years until the sequence of the AKH responsible in kissing bugs to control lipid metabolism became known (see 3.2 below, Reduvioidea).

Meanwhile, the first sequence of a member of the AKH family in a hemipteran insect was published in 1994 (13) from the South African orange wing cicada, *Platypleura capensis* (Superfamily Cicadoidea) in which two AKHs were isolated (**Table 1**). The two AKHs, called Placa-HrTH-I and -II, were also found in a variety of other cicadas by two further research groups (14, 15). The interesting fact here is that both peptides have an identical amino acid sequence and UV spectrum but differ in chromatographic retention time in liquid chromatography: peptide I elutes earlier than the more hydrophobic peptide -II. Edman degradation could not resolve the sequence difference at the time. Recent experiments suggest that the difference between the two peptides may be a cis-trans proline isomerization (17, 124). Functionally these peptides are not involved in lipid metabolism but are true hypertrehalosemic hormones as shown by the mobilization of carbohydrates *in vivo* in *P. capensis*, as well as by flight experiments where free flight of the cicada to exhaustion in about 8 min, decreased the titre of hemolymph sugar by 40% and the amount of glycogen in the flight muscles by 70% (13).

Historically the next hemipteran AKH was reported from the fire bug *P. apterus* (see below 3.2 Pyrrhocoroidea).

### 3.2 The Hemipteran AKHs: A Phylogenetic Context

#### 3.2.1 Suborder Sternorrhyncha, Superfamily Aleyrodoidea

Neuropeptide precursors and their receptors were identified in a specific strain of the whitefly *B. tabaci* (1) by analyzing the published whitefly genome and transcriptome (48) using sequence information from a set of five insects with previously characterized neuropeptidomes. In the process, an AKH receptor (AKHR) gene and an AKH precursor was identified. The latter represents a mature octapeptide AKH with the sequence pEVNFSPTW amide (**Table 1**). This peptide had been sequenced before *via* mass spectrometry as the first neuropeptide ever to have been structurally identified in the order Grylloblattodea specifically, in the ice crawler *Galloisiana yuasai*, hence, the code-name for this peptide is Galyu-AKH (125). Galyu-AKH is also identified from the transcriptome of a second white fly species, *Trialoeuodes vaporariorum* (**Table 1**), so that one can cautiously state that this peptide may be characteristic for Aleyrodidae. Although the sweet potato whitefly had been used in flight chamber experiments to study its ability of migration and dispersal (126), no flight metabolites had been measured. With the updated knowledge of its endogenous AKH and receptor, experiments could be undertaken to measure the extent to which lipids contribute to the energetics of dispersal flights.

#### 3.2.2 Suborder Sternorrhyncha, Superfamily Psylloidea

Transcriptomic data of the Asian citrus psyllid, *D. citri*, were analyzed to identify the core set of neuropeptides and their receptors based on homology with sequences from other insects (2). The AKH transcript revealed a sequence for a mature decapeptide AKH as pEVNFSPNWGG amide. A peptide with this primary structure is known by mass spectrometry sequencing in a member of the Order Embioptera, the webspinner *Oligotoma saundersii*, hence its code-name Olisa-AKH (127). Two AKHR genes were identified in the transcriptome of *D. citri* on the basis of homology (2), but these have not yet been characterized (functionally or pharmacologically) as being AKHRs. Spatial expression profiles revealed a rather surprizing ubiquitous appearance of the AKH gene in different body areas of the citrus psyllid (2). AKH mRNA is usually confined to the head of an insect, where the corpora cardiaca are located; it should be noted, though, that the expression profiles were generated with RSEM, a software tool for quantifying transcript abundances from RNA-Seq data by using an Expectation-Maximization algorithm to estimate maximum likelihood expression levels of a transcript. Moreover, *D. citri* adults are only 3 mm in size, thus increasing the handling difficulty and the accuracy of neatly separating body areas without tissue contamination. Similarly, the RSEM-generated expression profiles for the putative AKH receptors predicted low but ubiquitous expression of one of the receptors in *D. citri* and no clear pattern for the other AKHR (2). Nonetheless, the genomic data and available transcriptomes (2, 50) could be instrumental in further investigations that could provide information of specific metabolic regulation in psyllids. Because of its serious damage to citrus production as vector of a deadly bacterium, flight capability and dispersal potential have been studied quite well in the citrus psyllid, however, to our knowledge no experiments demonstrating the flight fuel for this insect have been undertaken. The transcriptomes of a further 8 species of Psylloidea from various families (**Table 1**) are publicly accessible: all have Olisa-AKH which appears to be the signature AKH for this superfamily.

#### 3.2.3 Suborder Sternorrhyncha, Superfamily Aphidoidea

The complete genome of *A. pisum* (52) as well as prediction of neuropeptides (54) and cloning of the AKH gene plus confirmation *via* mass spectrometry (6) all concluded in the same primary structure of a unique decapeptide, code-named Acypi-AKH (pEVNFTPTWGQa). The same sequence is found in at least another 19 species of aphids (**Table 1**), discovered mostly *via* molecular biology and bioinformatics methodologies. Biological assays with *A. pisum*, a 4 mm long adult aphid, are naturally challenging. It is not surprizing, therefore, that a classic metabolic effect (increased lipids, carbohydrates and/or protein) could not be measured in extracts of whole aphids after topical exposure to Acypi-AKH (6) and merely emphasizes methodological shortcomings arising from *in vivo* experimentation with such a small animal. However, enzyme assays could demonstrate an increase in the activity of glutathione-S transferase, an enzyme involved in antioxidative defensive mechanisms. Moreover, AKH transcript expression was chiefly in the brain/corpora cardiaca and ovaries/embryos, with significantly higher transcript numbers (calculated from quantitative real-time-PCR) in winged individuals compared with wingless morphs, interpreted as an indirect indication of the significance of AKH in aphid flight (6). In the eusocial aphid species, *Pseudoregma bambucicola*, the AKH receptor was cloned additionally, and three different splice forms found (7). A similar expression pattern in AKH transcript was found in *P. bambucicola* as in *A. pisum*, with the highest AKH expression level recorded in winged females and soldiers, and the lowest in wingless sedentary females (7). AKHR transcripts were located in gut, ovary, fat body and head of *P. bambucicola* with the highest levels in soldiers in galls and in winged females (7). Possible (unconvincing) explanations are put forward for the AKH/AKHR expression in winged/non-winged forms and different aphid castes and attempts to link it with various energy requirements (see 7).

As early as 1961 a quite detailed study by (128) investigated the relationship of the flight fuels glycogen and fats to tethered flight duration in *Aphis fabae*. During the first h of flight fats contributed about 70% to the used energy by the flight muscles, whereas after 4 h and 6 h it was more than 90%. Thus, without doubt, fat oxidation is preferred for long flights. This may be true for all other aphids as well. For example: tethered flight in a wind tunnel has been used for the corn leaf aphid *Rhopalosiphum maidis* to measure their ability to migrate and disperse (129). Best flyers were young, winged adults (half a day to one day old) that had about 50% of their dry weight measured as lipids. Flight could last for up to 14 hours with a sharp decrease of lipids during the first 4 h (a drop from about 50% to 25% dry weight) (129). Thus, we can conclude that the known AKH/AKHR system in *A. pisum* and other aphids plays a major role in energy provision and hope that this will stimulate experiments in which dsAKH/dsAKHR is introduced into the aphids and correlated with flight substrate use after flight episodes to obtain definitive answers about the relative importance of lipid vs carbohydrate metabolism in aphids.

#### 3.2.4 Suborder Sternorrhyncha, Superfamily Coccoidea

AKH data are only available from transcriptomes; our homology searches revealed that 11 out of 12 investigated species contain a novel decapeptide (Novel 1: pEVNFSPYWNQ amide) which structurally is an elongation of an octapeptide found in an aquatic heteropteran insect (Letin-AKH: pEVNFSPYW amide, **Table 1**). One coccineal species, has a S5 to T5 modification of the common decapeptide found in this superfamily; this too, is a novel peptide and we refer to this second novel peptide as Novel 2 (**Table 1**).

#### 3.2.5 Suborder Coleorrhyncha

The mossbugs are represented by 4 species in **Table 1**; their transcriptomes were assembled 4-8 years ago and our homology searches revealed the octapeptide Manto-CC (pEVNFSPGW amide) in 3 of the transcriptomes and the octapeptide Corpu-AKH (pELNFSPSW amide) in the remaining transcriptome (**Table 1**). Manto-CC had earlier been sequenced *via* mass spectrometry in a member of the polyneopteran order Mantophasmatodea (130), while Corpu-AKH is known from a corixid water bug (see below).

#### 3.2.6 Suborder Auchenorrhyncha, Superfamily Fulgoroidea

Genomic and transcriptomic analyses led to the prediction of one octapeptide AKH with the code-name Peram-CAH-I (pEVNFSPNWa) in the brown planthopper *N. lugens* (Family Delphacidae) (10, 11). This peptide is one of the two signature AKHs with cardioacceleratory and hypertrehalosemic action of blattid cockroaches but is also known to be synthesised by the CC of other insects including a few beetle families (21, 131). There are numerous other genomes/transcriptomes assembled and published from various families of Fulgoroidea; we found hits to our homology searches in 23 species (**Table 1**). Like in *N. lugens*, Peram-CAH-I is identified in 3 other Delphacidae species. For the rest, the majority of species, irrespective of family, has the octapeptide Anaim-AKH (pEVNFSPSW amide) which was first identified in a dragonfly *Anax imperator* (21); 2 species of the family Fulgoridae have Corpu-AKH, which we encountered also in the moss bugs; and the single accessible transcriptome of species of the families Tettigometridae and Tropiduchidae respectively, have Pyrap-AKH (pELNFTPNW amide), which was first found in the heteropteran firebug *Pyrrhocoris apterus*, and a novel decapeptide (Novel 3: pEVNFTPGWGG amide) which is an elongated form of an octapeptide (Psein-AKH) first sequenced from a damselfly (21).

Flight and metabolic fuels in species from the superfamily Fulgoroidea have only been studied in the 4.5 mm long planthopper *N. lugens*. That lipid is fuelling flight in *N. lugens* was investigated already in the 1980s. Not only was a depletion of the lipid stores noted after some flight time (well in excess of 20 min), but the lipid content determined from whole planthoppers at different points along their migratory routes decreased the further the individuals were away from their breeding sites, indicating a role of lipids as energy source during migratory flights (132). A role for carbohydrates as additional, minor flight fuel in *N. lugens* was concluded from another set of flight and metabolism studies (133): although lipid deposits are proportional to body weight and the glycogen reserves are more variable and approximately only 0.2% of the live weight of adult planthoppers, *N lugens* utilized the carbohydrate reserves at the beginning of their migratory flights for some hours before the total switch over to lipid reserves. When the AKHR cDNA of *N. lugens* was cloned (134), expression studies (using qRT-PCR) could be carried out and correlated with specific physiological conditions, including lipid metabolism. Expression levels of AKH transcripts were highest in adult male *N. lugens* and exclusively expressed in head tissues, while for the *N. lugens* AKHR the highest expression was in the fat body; the AKH/AKHR signaling system is most developed in the adult stage, as judged by quantitative expression during different stages of development (134). Starvation of between 6 and 48 h resulted in a significant increase in expression of AKHR transcripts, while AKH expression levels showed a significant difference at 48 h, suggesting a role for AKH signaling in starvation resistance and, indeed, injection of Peram-CAH-I (2 injections of 20 pmol per day for 3 days) into starving females saw a significant decrease and depletion of TAG stores in the fat body as this was mobilized in the form of DAG into the hemolymph to satisfy the metabolic needs of the body cells under nutrient deprivation. Such AKH-injected females, not unsurprisingly, succumbed faster to starvation (following the depletion of lipids in the fat body) than starving females that were not injected with an AKH (134). A similar delay in death by starvation was achieved when the AKH signaling system was knocked down in the brown planthopper – the AKH receptor was silenced by injection of dsRNA into female adult bugs: in such AKHR knockdown *N. lugens* females, obesity resulted along with an excessive accumulation of TAG in the fat body and a reduction of DAG in the hemolymph when compared with control-injected (ds green fluorescent protein) planthoppers. Moreover, these AKHR knockdown insects lived 24 h longer under starvation conditions than the control-injected *N. lugens* specimens (134), indicating that an intact AKH signaling system will continue to try and restore lipid homeostasis and consequently, deplete resources at a faster rate under starvation conditions. It is believed that a second, functionally redundant pathway of lipolysis (i.e. Brummer lipase-mediated) may be partially compensating for the impaired AKH signaling pathway in AKHR knockdown insects during starvation. Brummer lipase was shown to regulate lipid mobilization and starvation resistance in *N. lugens* (see 134). In a follow-on study with female *N. lugens* and the silenced AKHR, a decrease in trehalose in the hemolymph was observed (135) since carbohydrates were used but could no longer be replaced from glycogen stores in the fat body due to the defunct AKHR. Moreover, phenotypic changes were recorded that matched those when the vitellogenin receptor (VgR) had been knocked down, viz. delay in oocyte maturation, reduced Vg incorporation into the oocytes, reduced fecundity and a prolonged pre-oviposition period; this seemed to be a clear link between the AKHR and vitellogenesis. Western blot analyses of whole AKHR knockdown females, however, revealed that Vg synthesis in the fat body was not affected, whereas significantly reduced VgR levels were measured in the ovary (135). When 2 M trehalose was injected into the AKHR knockdown *N. lugens* specimens, the VgR gene expression and VgR protein levels in the ovary increased, and the incorporation of Vg into oocytes could be partially restored (135), suggesting either that AKH-mediated trehalose is involved in regulating vitellogenesis and oocyte development in the brown planthopper, or that the knockdown phenotype may have been rescued by the general provision of an energy substrate.

#### 3.2.7 Suborder Auchenorrhyncha, Superfamily Cicadoidea

The AKHs of 5 cicada species were sequenced by Edman degradation in the 1990s; the strange twin peptides, Placa-HrTH-I and -II, were discussed above (in **3.1**) with the research into metabolism - these cicada species utilize and mobilize carbohydrates, not lipids for flight. From accessible transcriptomes we have located AKHs of a few more cicada species (**Table 1**): 6 of the 7 additional species produce the decapeptide Placa-HrTH (pEVNFSPSWGNa) which is an extended form of Anaim-AKH, that is the hallmark peptide of the Fulgoroidea (Auchenorrhyncha). One of these 6 cicada species (*Maoricicada tenuis*), however, produces a second AKH in addition to Placa-HrTH - a novel decapeptide (Novel 4: pELTFSPSWGN amide), and only this novel peptide occurs in *Kikihia scutellaris* (**Table 1**).

#### 3.2.8 Suborder Auchenorrhyncha, Superfamily Cercopoidea

The structure of two AKHs from the CC of the spittle bug *Locris arithmetica* were fully characterized by mass spectrometry (16). One is the octapeptide called Peram-CAH-I (pEVNFSPNW amide) that we encountered and discussed with Delphacidae species (Auchenorrhyncha, Fulgoroidea); the other spittle bug AKH is the octapeptide code-named Pyrap-AKH (pELNFTPNW amide), which was first identified in the firebug *P. apterus* but is also synthesized in a pamphagid grasshopper and in the red flour beetle *Tribolium castaneum* (21). *In vivo* bioassays with *L. arithmetica* revealed that both peptides significantly raised the concentration of lipids in the hemolymph (and to a lesser extent, the level of carbohydrates). Moreover, metabolite changes in the hemolymph of the spittle bug during 2 min free flight episodes suggested that contractions of flight muscles are initially fuelled by the oxidation of carbohydrates but subsequently by lipid oxidation (16). The black and red frog hopper *Cercopis vulnerata* produces two very similar octapeptide AKHs: Peram-CAH-I was shown by mass spectrometry (17), and we identified Emppe-AKH (pEVNFTPNWa) from the frog hopper’s transcriptome (**Table 1**). Emppe-AKH had previously been found in the CC of the praying mantis *Empusa pennata* and later from six further species of the order Mantodea (136). Bioinformatic searches revealed a pair of AKHs in a third cercopoid species, *Prosapia bicincta*, viz. Peram-CAH-I and Psein-AKH, a damselfly peptide (**Table 1**). *C. vulnerata* and its close relative, the meadow spittle bug *Philaenus spumarius*, perform impressive and powerful jumps (60, 61, 63) but AKH regulation of substrate usage in these species has not been investigated to date. The study of AKHs from the African froghopper *Ptyelus flavescens* (Family Aphrophoridae of the Cercopoidea) elucidated three AKHs (18): an octapeptide, Peram-CAH-I, and two decapeptides, Ptyfl-AKH-I and -II, which differ only in one amino acid at position 7 (**Table 1**). *P. flavescens* only undertake short distance flights and, accordingly, biological assays showed mobilization of carbohydrates after injection of the endogenous peptides in synthetic form (18). The European alder spittlebug, *Aphrophora alni*, also infamous for extraordinary jumping abilities (63), contains a pair of octapeptide AKHs: Emppe-AKH was evidenced in a CC extract with mass spectrometry (17), while the near-similar Peram-CAH-I was deduced from database searches (**Table 1**). Only Peram-CAH-I could be identified as AKH in *P. spumarius* (**Table 1**). There are no metabolic data available on fuel use during flight or jumps for *A. alni* and *P. spumarius*.

#### 3.2.9 Suborder Auchenorrhyncha, Superfamily Membracoidea

One octapeptide, Anaim-AKH, was sequenced from the green leafhopper *Cicadella viridis* and the rhododendron leafhopper *Graphocephala fennahi* (17). Anaim-AKH was first identified in the dragonfly *A. imperator* (Order Odonata) (21) and is prevalent in the Fulgoroidea bugs (discussed above) but also in a number of water bugs (see below), as well as in members of the order Ephemeroptera (137). Leafhoppers are good flyers but have not been investigated in depth, especially not with respect to fuel utilization; instead, their dispersal flight abilities have been studied on flight mills, for example, the aster leafhopper *Macrosteles quadrilinaeus* (138) and *Cicadulina*, vector of the maize streak virus (139). Another notorious leafhopper pest insect that is under the spotlight is the glassy winged sharpshooter, *Homalodisca vitripennis*, which feeds on a variety of plants, *inter alia* grapes, stone fruits and citrus, into which it transmits the bacterium *Xylella fastidiosa* (20).

A wealth of AKH sequence information is derived from recent transcriptomic work on Membracoidea species, spanning various families (Cicadellidae, Membracidae, Melizoderidae, Myerslopiidae and Aetalionidae; **Table 1**). In all but one species of the Membracoidea listed in **Table 1**, only one AKH peptide is present or predicted. The exception to this rule is *Hespenedra chilensis* with two predicted AKHs. By far the most common AKH in this superfamily is Anaim-AKH (33 species in all), there are 4 species with Peram-CAH-I, two with Libau-AKH (pEVNFTPSW amide; first found in anisopteran dragonflies) (21) and a novel octapeptide (Novel 5: pEVNFTPYW amide) which has a S7/Y7 exchange compared to Libau-AKH, co-occurs with Libau-AKH in *H. chilensis*.

#### 3.2.10 Suborder Heteroptera, Superfamily Gerroidea

To date only one species of the long-winged water strider family Gerridae, *Gerris lacustris*, has been investigated for AKHs and an octapeptide (pEVNFSTGW amide) was sequenced (21). This peptide, code-named Grybi-AKH, was found in *Gryllus bimaculatus* and other ensiferan Orthoptera but is almost ubiquitous with presence in some caeliferan Orthoptera, Hymenoptera, Neuroptera and Dermaptera (21). Transcriptomic data confirm this interpretation and three more Gerroidea have been assigned with Grybi-AKH (**Table 1**).

Migratory or dispersal flights are quite common in Heteroptera, in general. Peculiar for this clade, the sclerotized forewings, the hemelytra, protect the membranous hindwings and, in flight are coupled with the hindwings to beat simultaneously (140). This is called functional diptery and it is necessary because there are no flight muscles associated with the hindwings. Although water striders are known for migratory flights, see for example (141), there are no reports on AKH-regulated energy metabolism. There is a general study on the lipids in the species *Gerris remigis* during season and development; it is stated that during hibernation “they probably metabolized 0.02 mg lipid/individual per day” (142); no involvement of an AKH has been noted.

#### 3.2.11 Suborder Heteroptera, Superfamily Corixoidea

The water boatman, *Corixa punctata*, contains one octapetide AKH with the sequence pELNFSPSW amide which was first elucidated in this species and, thus, is called Corpu-AKH (22). As discussed above, Corpu-AKH is now also predicted in a Coleorrhynacha and two Auchenorrhyncha species. Conspecific bioassays clearly showed that hemolymph lipids were elevated upon injection of synthetic Corpu-AKH and not the carbohydrates, suggesting a lipid-based metabolism (22). Corixidae fly and swim very well (96, 143) but contrary to other water bugs (see below) experimentation into the usage of metabolites during such activity is lacking.

#### 3.2.12 Suborder Heteroptera, Superfamily Nepoidea

AKHs of six species of this suborder have been sequenced, 3 from the family Nepidae and 3 belonging to the family Belostomatidae. All of these AKHs are octapeptides: two are found only in this superfamily, viz. Nepci-AKH (pELNFSSGW amide) in the water scorpion *Nepa cinerea* and Letin-AKH (pEVNFSPYW amide) in the giant water bug *Lethocerus indicus* (23), while two other Nepidae (*Ranatra linearis* and *Laccotrephes fabricii*) produce Grybi-AKH and Peram-CAH-I, respectively (23, 24); *Hydrocyrius columbiae* (Belostomatidae) synthesizes two AKHs in the CC, viz. Letin-AKH and Anaim-AKH, while only Anaim-AKH is produced in *Appasus grassei* (Belostomatidae) (24). Conspecific bioassays showed for *N. cinerea*, *L. niloticus*, *A. grassei* and *L. fabricii* an increase of the hemolymph lipids when injected with the respective endogenous AKH but no change in the titre of carbohydrates, indicating the involvement of lipids in energy metabolism (23). This was corroborated by experiments where *A. grassei* or *L. fabricii* specimens had to swim continuously for one hour, thus perform strenuous exercise. The release of AKH, activation of TAG lipase in the fat body and DAG release into the hemolymph is indirectly shown by the substantial increase in the titre of lipids after the exercise (24).

#### 3.2.13 Suborder Heteroptera, Superfamily Ochteroidea

We have no other data to present here, except that we identified Anaim-AKH from the published transcriptome of *Gelastocoris oculatus* (Family Gelatocoridae; **Table 1**).

#### 3.2.14 Suborder Heteroptera, Superfamily Naucoroidea

The AKHs of three members of this superfamily are known: Anaim-AKH is present in the European saucer bug *Ilyocoris cimicoides* (Family Naucoridae; 22) and in the permanently submerged water bug *Apheilocheirus aestivalis* (Family Aphelocheiridae; 25), while a superfamily-specific C-terminally elongated form of Anaim-AKH, called Lacsp-AKH (pEVNFSPSWGG amide), occurs in the African saucer bug *Laccocoris spurcus* (25). Homospecific *in vivo* bioassays in which Naucoroidea bugs were injected with a synthetic species-specific endogenous AKH always resulted in a hyperlipemic not hypertrehalosemic response (22, 25). Moreover, during physical activity of these two saucer bugs, such as swimming for 1 h, the concentration of lipids in the hemolymph was significantly elevated up to 2-fold (22, 25). The experimental data, thus, indicate a lipid-dominated energy metabolism.

#### 3.2.15 Suborder Heteroptera, Superfamily Notonectoidea

The AKH of three species of the same genus, *Notonecta*, was characterized by mass spectrometry as the octapeptide Anaim-AKH (26, 27). Transcriptomic information from a fourth notonectid bug had Anaim-AKH as well (**Table 1**). There is plenty of information that Notonectoidea bugs have a lipid-based metabolism. The lipid concentration in the hemolymph of resting *N. glauca* was about 5- to 7-fold higher than the carbohydrate concentration; also, the glycogen content in the thorax (mostly flight muscles) and abdomen (mainly fat body) was very low and would be insufficient to sustain prolonged or intense metabolic activity during energy-intensive locomotion, such as flight (26). *Notonecta* species are known for their flight abilities either to avoid conditions of desiccation in temporary water bodies or to undertake dispersal flights from pond to pond for reproductive reasons (144). Metabolites and locomotion (flight and swimming) were studied in *N. glauca* (26): an hour of rest following a 3 min uninterrupted flight episode nearly doubled the lipid concentration in the hemolymph, indicating that lipids were mobilized for flight and remained high during the resting period with no muscular activity to consume the flight fuel; a low level of carbohydrates contributed negligently to power output. Constant swimming for 1 h similarly increased the circulating levels of lipids significantly. Another piece of evidence that lipid oxidation is the major form of energy production in this species came from conspecific bioassays. Injection of CC extracts only caused hyperlipemia and no change of hemolymph carbohydrates. Injection of as low a dose as 1 pmol of synthetic Anaim-AKH into resting *N. glauca* was sufficient for a near doubling of the lipid titre in the hemolymph (26).

#### 3.2.16 Suborder Heteroptera, Superfamily Saldoidea

The transcriptome of one species of this superfamily revealed an AKH with the structure pELTFSPSW amide, code-name Tippa-CC-II, which had been identified by mass spectrometry in a tipulid fly (145).

#### 3.2.17 Suborder Heteroptera, Superfamily Reduvioidea

As the genome of *Rhodnius prolixus* was being assembled and annotated, selected access to the data allowed a prediction of an octapeptide AKH in this key vector of Chagas disease 4 years before publishing the genome (28). The prediction was confirmed when Rhopr-AKH (pELTFSTDW amide) was sequenced from *R. prolixus via* mass spectrometry, along with the AKHs from three other triatomine kissing bugs (29): *Panstrongylus megistus* also produces Rhopr-AKH, while *Triatoma infestans* produces a nonapeptide (Triin-AKH: pELTFTPNWG amide) and *Dipetalogaster maxima* a decapeptide (Dipma-AKH: pELTFSDGWGN amide). To date, two of the three AKHs of the kissing bugs are unique for Hemiptera – Triin-AKH occurs also in Lepidoptera (89).

Thirteen years after Ward et al. (123) had studied flight metabolism of *R. prolixus* and *T. infestans* (see above **3.1**), clear evidence was obtained that lipid metabolism is important in the triatomine bugs *P. megistus*, *D. maxima* and *T. infestans* (146). Following injection of heterologous AKHs into the bugs, a low density lipoprotein was detected in the hemolymph – exactly as is known from the action of AKHs in locusts, where AKH causes a reorganization of hemolymph proteins and lipoproteins to form low-density lipoproteins (to which DAG is loaded), increasing the lipid-carrying capacity (115, 147). Although Ward’s investigation did not include flight as a parameter (123), this was “remedied” 8 years later when flight in *P. megistus* was investigated and it was shown that only a small amount of carbohydrates was used during the initial flight period and mostly lipids contributed to longer flights (148) Furthermore, resting *P. megistus* specimens had only high density lipophorin in the hemolymph, whereas low density lipophorin was detected after flight thus, the lipophorins were loaded with diacylglycerols made available from the fat body under the mobilizing action of AKH.

Biological assays in which *R. prolixus* (5^th^ instar larvae or adults) were injected with synthetic Rhopr-AKH resulted in significant lipid accumulation in the hemolymph (29, 149). Moreover, injection of the endogenous AKHs of *T. infestans* and *D. maxima*, respectively, also had a hyperlipemic effect (29). Immunocytochemistry localized AKH-producing cells solely as neurosecretory cells of the CC of *R. prolixus* and suggests that AKH is released from this neurohemal organ into the hemolymph (149). The complete cDNA of the AKH (150) and of the AKH receptor (150, 151) of *R. prolixus* were cloned and sequenced. The Rhopr-AKH gene was only expressed in the CC of 5^th^ instar and adults but not in the central nervous system (150), while the receptor transcript is mainly found in fat body and flight muscle and to some extent in the dorsal vessel (150, 151). It must also be stated here that the sequence for the *R. prolixus* AKH receptor presented in the publication by Ons et al. (152) is not correct. Clear evidence for this is also the lack of finding a close relatedness between *P. apterus* and the Rhopr-AKHR (using the sequence given in 152), whereas other insect AKHRs are well related (153).

The involvement of the AKH signaling system in lipid metabolism was established in *R*. *prolixus* (150, 151). Knockdown of the Rhopr-AKHR gene resulted in either TAG accumulation in fat body and flight muscle after starvation (151) or an increase in the level of abdominal ventral lipid content with a simultaneously lower lipid level in the hemolymph (150). The latter study also showed that lipid titres in the hemolymph were not elevated after injection of Rhopr-AKH in receptor-knockdown insects. Alves-Bezerra et al. (151) also researched the question how AKH signaling can affect TAG levels. Knockdown of Rhopr-AKHR resulted in a small but significant increase of transcripts of the enzyme acyl-CoA-binding protein1 (the enzyme that is necessary for acyl-CoA transport) and a big decrease of transcripts for the enzyme mitochondrial-like glycerol-3-phosphate acyltransferase; both enzymes are required during the synthesis of TAG. Transcripts of these enzymes were also induced by injection of Rhopr-AKH, leading to the hypothesis that AKH signaling in *R. prolixus* may be necessary for the regulation of nutrient metabolism by changing the expression profile of target genes.

The possible role of AKH signaling in carbohydrate metabolism in female *R. prolixus* was recently reported (154): trehalose content increased in the fat body and ovaries following a blood meal, as did the circulating levels of trehalose in the hemolymph; this coincided with the start of vitellogenesis and a significant increase in the transcript expression of a sugar transporter, specifically a trehalose-facilitated transporter (Rhopr-TRET), whereas the transcript expression of enzymes involved in trehalose synthesis was no different to that of unfed kissing bugs. Thus, TRET could be facilitating bidirectional transfer of trehalose in the fed state, ie. the release of trehalose from the fat body and the uptake by the ovaries for egg formation. Assays using insulins and Rhopr-AKH further showed that *Rhopr-TRET* was upregulated in the ovaries by both peptide families during vitellogenesis. In the unfed condition, Rhopr-AKH signaling might be involved in the release of trehalose from the ovaries *via* Rhopr-TRET, thereby providing a source of energy for the insect during stressful situations such as starvation (154). Further experiments are required to fully unravel the role of AKH in regulating trehalose metabolism in triatomine bugs.

#### 3.2.18 Suborder Heteroptera, Superfamily Cimicoidea

When the genome of the bedbug C. *lectularius* was published (75, 76), it was only a question of time until the neuropeptidome was identified and made public (30). An octapeptide AKH with the sequence pEITFSTGW amide was predicted and shown to exist by mass spectrometry. This previously unnamed peptide was also found by genome mining of the red harvester ant, *Pogonomyrmex barbatus* (order Hymenoptera) (155, 156) and, here, we code name it: Cimle-AKH. Transcriptome mining of other members of the Cimicoidea, *Orius insidious* and *O. laevigatus* (Family Anthocoridae), displayed a close relative to Cimle-AKH in both bugs with a I2/L2 exchange; such a molecule was previously known to occur in a Lepidoptera with the code-name Dircl-AKH-II (89). Bed bugs are wingless, thus there are no studies on metabolic regulation during any form of exercise.

#### 3.2.19 Suborder Heteroptera, Superfamily Miroidea

The Miroidea seem to be a special case. There are quite a number of transcriptomes known and the putative mature AKHs are almost all different. In the western tarnished plant bug *L. hesperus* (Family Miridae) two related octapeptide AKHs occur that differ only at position 7 (Novel 6 and 7: pELTYSPG/NW amide, **Table 1**) and are unique for Hemiptera (32). Flight experiments on a flight mill were conducted with *Lygus lineolaris* (98) and *Lygus lucorum* (scientific name: *Apolygus lucorum*, Meyer-Dür 1843) (157) with quite similar results: young reproductive female bugs make longer (migratory) flights and “are more likely to colonise new habitats” than any other age or sex group investigated. When *A. lucorum* were tested for 48 h continuously on the flight mill, individuals flew roughly 70 km with a maximum of 150 km, thus, true long-distance migratory flights. To date, unfortunately, biochemical studies into fuel usage during flight have not been undertaken. The tools to study hormonal regulation are now available for this family. We present (**Table 1**) AKH sequences that we found from BLAST searches of 10 Miridae representatives: apart from *L. hesperus* with two novel AKHs (32), *A. lucorum* is the only other species that has two AKHs, viz. Glomo-AKH and Dircl-AKH-II which are known from Diptera (145) and Lepidoptera (89), respectively. A further 3 species have a novel octapeptide (Novel 8: pELTYSTNW amide), while a novel extended form of Novel 8 is present as a nonapeptide (Novel 9: pELTYSTNWG amide) in another species, and yet another species has the Novel 6 AKH that is also present in *L. hesperus* (**Table 1**). Of the remaining 3 species, 2 produce Peram-CAH-II and 1 has a novel dipteran AKH that we had first reported on in the family Diopsidae and in *Drosophila grimshawi* (pELTFSPNW amide) (145). In conclusion, the Miridae are a total “mixed bag” with respect to AKH structures, and it is possible or even very likely that more structures can be found in this superfamily, since for one reason or another AKH evolution has flourished in this clade.

#### 3.2.20 Suborder Heteroptera, Superfamily Pentatomoidea

The first report on the primary sequence of an AKH in a member of the Pentatomoidea showed an octapeptide synthesized in the CC of the southern green stinkbug *Nezara viridula* (34). The surprizing and spectacular finding was that the sequence was identical to the well-known, conserved decapod crustacean red pigment-concentrating hormone (RPCH), Panbo-RPCH (pELNFSPGW amide). Thus, Panbo-RPCH is not exclusively present in Crustacea as previously thought. Today, we know that Panbo-RPCH occurs also in a beetle species and is the only AKH known from the Order Plecoptera (158). Its presence in various other species of stinkbugs of the family Pentatomidae was confirmed (**Table 1**), whereas a member of the family Acanthosomatidae had the octapeptide Schgr-AKH-II and a family member of the Tessaratomidae had two octapeptides, Panbo-RPCH and Schgr-AKH-II (27). A second minor AKH (about 10% of the amount of Panbo-RPCH) was identified in *N. viridula* as a post-translationally modified peptide: instead of proline in Panbo-RPCH a hydroxyproline residue at position 6 occurs in Nezvi-AKH (35). There are a number of publications on flight ability, flight distance of sexes at different ages, involvement of diapause, to name a few parameters for *N. viridula* (103) and also for the marmorated stinkbug *Halyomorpha halys* (101) on a flight mill. Some biochemical/physiological parameters with respect to flight fuels and AKH regulation are known from *N. viridula* and the inflated stinkbug *Encosternum delegorguei* (16). In *E. delegorguei* the lipid concentration in the hemolymph is 10-fold higher than the carbohydrate concentration, in *N. viridula* only 2-fold. Lift-generating flight of 3 min increased the lipid titre in *E. delegorguei* slightly and even more 1 h rest post flight; carbohydrate titre decreased upon flight and was back to pre-flight levels one hour resting after the flight episode. Similar results were achieved with *N. viridula*: here it could be shown that longer flights (10 min) resulted in a greater accumulation of lipids in the hemolymph than short flights of 2 min, whereas carbohydrates were nearly diminished after 10 min of flight; during this time glycogen phosphorylase in the fat body was clearly activated. The amount of glycogen stored in thorax and abdomen of *N. viridula* is very small compared to the stored lipid content. On the other hand, biological assays in which the endogenous AKH is injected into the green (34) or inflated stinkbug (16) resulted always in hyperlipemia but not hyperglycemia/-trehalosemia. It appears, thus, that upon flight AKH is released, activates a phosphorylase and the small amounts of carbohydrates stored in hemolymph and fat body are used at the beginning of the flight and thereafter only at a low rate, whereas a lipase activation makes use of the larger stores of lipids. This pattern is very reminiscent of fuel usage during flight in locusts (see 159).

Flight duration of *N. viridula* in relation to environmental and biological variables were studied using tethered insects (103). Under the non-lift flight conditions, insects could fly for longer than 10 min. The flight duration of the bugs, which was measured as a function of post-ecdysis age, peaked on the eighth day and thereafter declined significantly with age. There was no difference in flight capacity between males and females, but flight durations were significantly different between virgin and mated bugs at ages from six to 12 days after ecdysis, with more virgin bugs than mated ones flying for extended periods. In another experiment, food deprivation and withholding of a mate increased the flight durations of males and females. Increasing temperature (20 to 30°C), however, retarded the flight ability in both diapausing and non-diapausing bugs, and none of the individuals tested flew for 30 min or longer at a temperature of 30°C (103); this is quite interesting, given that *N. viridula* and other stinkbug species are active in the austral summer where temperatures are well above 20 deg C on a daily basis and that captured bugs flew more readily at higher temperature in our Cape Town laboratory (16). Field observations suggest that the green stink bug may make massed migratory flights before diapause, and it seems that females may migrate as virgins (103).

#### 3.2.21 Suborder Heteroptera, Superfamily Pyrrhocoroidea

The first AKH in a heteropteran Hemiptera was elucidated from the CC of the firebug *Pyrrhocoris apterus* as the octapeptide Pyrap-AKH (160). In biological assays this peptide activated lipid mobilization and stimulated general locomotory behaviour/activity. Although the authors “excluded the possibility of the presence of other peptides with adipokinetic activity” (160), two years later they unambiguously identified a second octapeptide with lipid-mobilizing activity in *P. apterus* from its CC - the well-known peptide Peram-CAH-II (**Table 1**) from blattid cockroaches (21), which differs from Pyrap-AKH only at position 3 by a T/N exchange (161). Investigations into which lipid species were mobilized by each of the two firebug AKHs, using HPLC and MS, demonstrated that DAGs are selectively mobilized from the fat body into the hemolymph: 4 DAGs consisting mostly of C16 and unsaturated C18 fatty acids were observed and the relative levels of 3 these DAGs were significantly different following the specific action of either Pyrap-AKH or Peram-CAH-II, thereby suggesting that a particular AKH mobilizes a particular fatty acid (162). The reason for this preferential mobilization is not clear but it was previously observed in the locust *L. migratoria* (163).

Since the firebug is easy to rear in the laboratory and had been a “convenient experimental model” for years (160), Kodrik and co-workers examined the theme “AKH and *Pyrrhocoris*” since two decades under various scientific aspects. First, an enzyme-linked immunoassay (ELISA) was developed to measure the amount of AKHs in various parts of the nervous system and in circulation (164), and this tool could be used to address various questions about AKH release dynamics, lipid metabolism and interaction with harmful molecules and parasites (e.g. pesticides), and metabolic differences between long-winged and short-winged morphs. The specificity of the AKH ELISA in *P. apterus* was obtained with a primary antibody that specifically interacted with the four C-terminal amino acids of the two endogenous peptides, i.e. TPNW amide. Appreciable amounts of AKH were only found by ELISA in the CCs, about 4 pmol per pair. For titre determination in the hemolymph pre-purification steps were necessary, making this method tedious, cumbersome and not user friendly for the general scientific community. Nevertheless it was used by the ELISA developer and about 1 fmol AKH/µl was measured in the hemolymph of quiescent reproductive female linden bugs (164). Tissue immunocytochemistry with the same primary antibody that was used in the AKH ELISAs revealed strong cellular staining in the CCs, along with faint staining in a few cells and axons in the brain (165). Although *P. apterus* adults are flightless, they display wing-polymorphism, i.e. macropterous (long-winged) and brachypterous (short-winged) forms occur; the highest locomotory activity (walking) was observed in macropterous morphs and they displayed a greater adipokinetic response than their brachypterous counterparts. This then raised the question whether/how AKH content in brain/CC and titre in the hemolymph was changing in the different morphs during development and over a 24 h daily cycle (165). In general, the AKH content in the central nervous system (CNS) increased slightly during development up to 14 days of adult life and macropterous females always displayed the significantly highest amount of AKH with about 4 pmol/CNS; the titre in the hemolymph was mostly below the detection limit of the ELISA but when detectable, mostly macropterous females had measurable titres of about 1.2 fmol/µl. Thus, the increased locomotion and dispersal of macropterous bugs are positively correlated with released AKH and more intensive mobilization of lipids. Variation of the AKH content in the CNS and hemolymph of 10 days old macropterous females were also monitored during a 24 h period under long day conditions (18h L:6h D) where the scotophase was set from 1 h to 7 h. Diel changes in AKH concentration in the CNS (synthesis and storage site) were recorded and this differed with the diel changes monitored in circulation (released AKH): AKH concentration in the CNS peaked at 17 h and was lowest towards the end of the photophase around 21 h, whereas the AKH titre in the hemolymph was low to undetectable around the end of scotophase/beginning of light phase and highest (almost 4 pmol/µl) from 14 h to 24 h, thus the later part of the light phase. There was no correlation between endogenous circulating AKH levels and an adipokinetic response after injection of 10 pmol of a synthetic AKH (161), but this absence of an enhanced response is not entirely unexpected since the AKHR is sensitive to small amounts of AKH and 10 pmol had functioned maximally in *P. apterus* in this study. AKH titre was also increased in the hemolymph of macropterous and reproductive brachypterous firebugs when insects were stressed by various factors: 20 min of forced running activity by shaking, introducing insects for 20 min to a dark environment during light phase and applying various doses of the insecticide permethrin (166). When permethrin was co-injected with (a high dose of) the endogenous Pyrap-AKH, the mortality of *P. apterus* increased about 2.3-fold above injection with the insecticide alone, thus the AKH enhanced the effect of the insecticide (167). This enhancement effect could be explained by measuring CO_2_ production as indicator of the metabolic rate in treated bugs: the insecticide treatment alone increased the AKH titre significantly, with the expected effect of elevating the metabolic rate. Not unsurprizingly, the effect of exogenous AKH in tandem with the insecticide clearly raised CO_2_ production above the level measured in bugs treated only with insecticide, which led to the conclusion that the increased metabolism allowed rapid penetration and uptake of the insecticide into cells resulting in the observed mortality rate after dual treatment (167).

The AKH genes of *P. apterus* were cloned - the deduced AKH precursors are quite similar (168). *In situ* hybridization and quantitative reverse transcription PCR localized AKH expression (both AKHs) not only in the CC but also in the brain. A Blast-P search of the transcriptome of *P. apterus* and alignment with known AKH receptors was used to identify the firebug AKH receptor sequence which was then confirmed by PCR and sequencing (153). These authors made use of AKHR knockdown to show that AKH signaling played an enhancing role to induce mortality when firebugs were infected with the entomopathogenic nematode *Steinernema carpocapsae*, very likely *via* a similar mode of action as observed when exogenous AKH was applied together with an insecticide, viz. that the increase in metabolism brought on by AKH effectively amplifies the activity of the nematodal toxins with rapid exchange of metabolites.

AKHs were elucidated from a further 3 Pyrrhocoridae species (**Table 1**), all of them containing only one AKH, viz. Pyrap-AKH (27).

#### 3.2.22 Suborder Heteroptera, Superfamily Coreoidea

In three species of the family Coreidae and one from the family Stenocephalidae the octapeptide Schgr-AKH-II (pELNFSTGW amide; **Table 1**) was found present (27, 37). This AKH was first discovered in the desert locust *Schistocerca gregaria* and is also present in many caeliferan and ensiferan Orthoptera, as well as in Hymenoptera (21). The species *Holopterna alata* has a second AKH which occurs in about 10% the amount of Schgr-AKH-II. It is a post-translationally modified form of Schgr-AKH-II where the T6 residue is sulphated (38). This twig wilter was examined more thoroughly and the following physiological account emerged (37): TAG lipase in the abdominal fat body has a 7-fold higher activity than the enzyme in the flight muscle and it is activated significantly by low doses of the endogenous Schgr-AKH-II, whereas the flight muscle enzyme is not activated. Lipids had a higher concentration not only in the hemolymph but also in the thorax and abdomen. Upon flight, when AKH is supposedly released from the CC, the small amounts of carbohydrates present in the hemolymph and thorax (glycogen) are significantly diminished after a short flight of 3 min and TAGs are utilized in the thorax and abdomen, while DAGs increase as the only lipid class in the hemolymph in the resting period after flight. These data are convincing to attribute AKH-regulated fat metabolism to this species.

#### 3.2.23 Suborder Heteroptera, Superfamily Lygaeoidea

Two species of the family Lygaeidae have been investigated for their AKHs and both, the large (*Oncopeltus fasciatus*) and small (*Lygaeus kalmii*) milkweed bug have the octapeptide Tenmo-HrTH (pELNFSPNW amide) (39; Gäde G, Šimek P, Marco HG unpublished). This peptide was first identified in *Tenebrio molitor* and is present in most members of the superfamily Tenebrionoidea but also occurs in the family Polyphaginae of cockroaches (21, 131). All experimental evidence on *O. fasciatus* indicates a lipid-based metabolism: the concentration of lipids in the hemolymph is about 10-fold higher than the titre of carbohydrates; the abdominal fat body of females contain 6 mg of lipids but only a minute amount of glycogen (less than 40 µg); injection of physiological amounts of synthetic Tenmo-HrTH had a pronounced adipokinetic effect on hemolymph lipids; tethered flight of both sexes of the milkweed bug resulted in a 30 to 60% increase of hemolymph lipids (39).

### 3.3 Hemipteran AKHs: A Mixture of Known and Novel Structures

We were able to identify AKHs from 191 species by literature searches, bioinformatics (searching genomic/transcriptomic databases) and mass spectrometry. In total 42 different AKHs have been characterized by primary structure from Hemiptera. Of these, 28 are octapeptides, 2 nonapeptides and 12 decapeptides. 20 sequences are known to occur in other orders, as well and the remaining 22 sequences, including the post-translationally modified peptides with hydroxyproline, sulphated threonine or a putative cis-trans proline modification, are to date unique to the order Hemiptera. Nine of those are novel AKHs, meaning they have never been reported to occur in any insect: 4 octapeptides, 1 nonapeptide and 4 decapeptides. A comparison with two other species-rich orders, albeit from the holometabolous insects, Diptera and Lepidoptera, is quite revealing (89, 145): first, far fewer species have been analyzed for AKHs as at two years ago (100 Diptera and 77 Lepidoptera); second, these two orders have rather order-specific AKHs, meaning that only 2 out of 14 (Diptera) or 1 out of 17 (Lepidoptera) of the sequences occur in another order; third, whereas Diptera contain almost exclusively octapeptides (only one decapeptide), Lepidoptera produce 5 octa-, 4 nona- and 8 decapeptides.

On the other hand, what is remarkable is that especially in the basal hemipteran families we have a superfamily specificity of AKHs: i.e. ALL to date investigated whiteflies have Galyu-AKH; all psyllids have Olisa-AKH, all aphids have Acypi-AKH, and all Coccoidea have novel AKH 1 (or 2 which is only a S/T exchange). Is there a possible explanation for this distribution pattern in the various orders? One can only speculate, of course. It appears, that the Hemiptera being an advanced hemimetabolous clade could “incorporate” a high number of the AKHs that had evolved before in more basal orders including Polyneoptera, whereas in the holometabolous orders a second wave of “AKH evolution” started resulting in novel and mostly order-specific AKHs.

### 3.4 Attempt of a Molecular Evolution of Hemipteran AKHs

**Figure 2:**
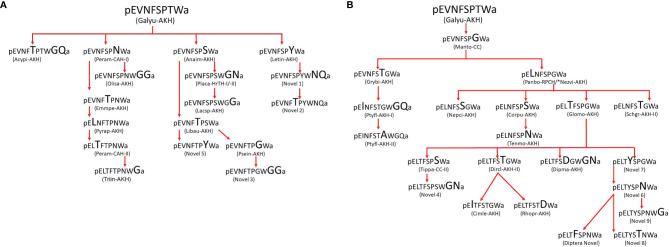
Hypothetical molecular evolution of adipokinetic peptides in Hemiptera. Galyu-AKH is assumed as ancestral peptide for this order: 5 AKHs can be directly derived from Galyu-AKH and *via* point mutations and elongation, the remaining 36 hemipteran AKHs can be derived. Due to space constraints, the figure is split into two panels **(A, B)**. The amino acid substitution or elongation in each peptide is indicated in a larger font than in the peptide from which it is hypothetically derived.

As outlined previously (89), one is always in a conundrum to decide on a putative ancestral AKH for this type of speculation. For the current study, we have selected the octapeptide Galyu-AKH, the AKH found in the two whitefly species (**Table 1**), which occurs in the phylogenetically most basal superfamily of the Hemiptera, the Aleyrodoidea. In so doing we may have a chance to discuss the speculative AKH phylogeny tree (constructed purely from molecular evolution of mature AKH sequences; **Figure 2**) with the existing phylogenetic knowledge of the Hemiptera (3; **Figure 1**). As depicted in **Figure 2** it is entirely possible to build such a tree for the possible molecular evolution of the AKHs of Hemiptera from encountered sequences in this order; there is no need for introducing hypothetical peptides but elongation events from octa- to nona- and decapeptides must have occurred. The question if this hypothetical scheme has anything to do with the reality, will be answered by future studies.

Looking at **Figure 2** purely from a phylogenetic point of view, it is interesting and pleasing to see that the majority of the AKHs known from the basal suborders, Sternorryncha and Auchenorrhyncha, are directly related to Galyu-AKH (viz. Acypi-AKH, Peram-CAH-I, Olisa-AKH, Anaim-AKH, Manto-CC) or have evolved from the “second row” of peptides. There are, of course, always exceptions to the rule: the heteropteran peptides Letin-AKH, Panbo-RPCH and Grybi-AKH are not at expected positions in the scheme (**Figure 2**) - or probably, we will find that these peptides also occur in basal clades once we have even more data. In general, however, it can be stated that molecular and phylogenetic evolution can be reconciled to a certain extent in the case of hemipteran AKHs.

### 3.5 Possible Lessons With Respect to Green Insecticides and Hemipteran AKHs

The rationale of “green insecticides” in integrated pest management is to use information about endogenous hormones of pest insects to make peptide mimetics that will act against the pest insects to alter their behaviour or physiology, while taking care not to harm beneficial insects, other organisms in the food chain, or the environment (111). Such “green” insecticides are designed, thus, on the basis of ligand–receptor interaction to have finally a mimetic at hand that is harmful only to pest insects. For this concept to work, one has to first identify the ligands and receptors in pest and beneficial insects, and secondly show specificity of action of natural ligands and mimetics, as well as demonstrate degradable and non-damaging effects on the environment.

In the current paper we review AKHs from the order Hemiptera. The most harmful hemipterans on an economic scale are aphids (Hemiptera: Sternorrhyncha: Aphidoidea). There are, additionally, several other hemipteran species that are pest insects. Can the current study with 42 AKH sequences from 191 hemipteran species provide any useful pointers on the potential of the “green” insecticide concept for Hemiptera AKHs? We see that there are 22 unique AKH peptide sequences, i.e. over 50% of the known hemipteran AKHs are only present in this order. Moreover, there seems to be some specificity at the superfamily level. It should be cautioned, though, that the sequences we obtained *via* bioinformatic searches in the present study have not yet been substantiated by mass spectrometry or peptide chemistry. This, notwithstanding, the number of unique sequences holds great theoretical potential to find a specific lead around which a mimetic could be designed with order-specificity. However, not all of the Hemiptera are deemed pests, and many are essential components in fragile ecosystems, such as the aquatic bugs. It would be imperative, therefore, to not only show group-specificity but also species-specificity of an AKH lead. For this, *in vivo* biological data is largely lacking in which the different AKHs have been tested in bug species, as for example done in Lepidoptera (89). Since many of the hemipteran pest insects are very small, conclusive evaluation at the receptor binding level will have to be performed to ascertain whether specificity is possible by using the various superfamily-specific bug AKHs as ligand for expressed pest insect receptors. This underscores the need for nucleic acid sequences of hemipteran AKH receptors.

We tested the AKHR of the pea aphid in *in vitro* assays with 4 of the hemipteran AKHs that co-occur in other insect orders, viz. Glomo-AKH, Schgr-AKH-II, Grybi-AKH and Peram-CAH-I, as well as with the endogenous Aphidoidea-specific AKH, i.e. Acypi-AKH, and found that the aphid AKHR readily accepted all of the AKHs bar one in the nanomolar range: activation with Schgr-AKH-II was only about 50% (169). On the other hand, Acypi-AKH did not bind to the AKHR of the honeybee, the vinegar fly, the yellow fever mosquito and the red flour beetle, and activated the desert locust AKHR and the pine weevil AKHR by only 30% (169). This demonstrates subtle activation differences of AKHRs, giving hope that specificity for aphids and other hemipteran pests may be established on a case-by-case basis.

## 4 Conclusions

We do know quite a bit about the most important regulator of lipid and carbohydrate metabolism, the AKHs, in Hemiptera. In future, even more sequences will be revealed because of the information assembled from whole genomes and from transcriptomes. Sequence information not only of the hormone but also its receptor is crucial, to have molecular information of the complete signalling system accessible for experimentation. This may be advantageous to investigate the usage of the AKH signalling system for purposes of designing next generation insecticides, especially for those insects that have agricultural, or horticulture or medical pest status as harmful herbivores or as vectors for all sorts of devastating microorganisms. In addition, one should encourage further physiological experiments in which AKH is upregulated, over-expressed or simply exogenously added in conjunction with other introduced deleterious factors (e.g. toxins from bacteria and fungi, or commercial pesticides), to assess how effectively we can exploit the natural metabolic actions of AKH to effect mortality in targeted, economically important hemipteran insects.

Not all of the Infraorders and superfamilies of Hemiptera are represented in our survey as we could not find any species of litter bugs (Dipsocoromorpha), unique-headed bugs (Enicocephalomorpha), semi-aquatic bugs (Gerromorpha: Mesoveloidea and Hydrometroidea), damsel bugs and flat bugs (Heteroptera: Naboidea and Aradoidea) to investigate ourselves, nor could we find any AKH sequences from these groups in publicly accessible databases. This is a gap that needs filling in the future to have a complete overview of the entire order. What is clear from our survey is that AKHs play a metabolic role in every species which has been studied in a bit of detail. When locomotory activity such as flight or swimming is involved, AKHs are surely responsible for the extra amount of ATP necessary to power the contracting muscles, by making lipids and finally, fatty acids available for oxidation. Since migratory dispersal flights are crucial for most pest insects, it seems to be a rewarding target to study the AKH signalling system further. This should extend also to flightless bug species and in bugs that are too small to handle in classic physiological experiments, as one could approach functional aspects *via* a molecular biology route. Information on unique primary structures is helpful in this regard.

## Author Contributions

Both authors contributed towards the research for and writing of the manuscript. GG conceptualized the review; HM co-developed the concept, performed bioinformatic searches and co-prepared Table and Figures, formatting and editorial checks. Both authors have approved the content of the manuscript and the submission thereof.

## Funding

This work is based on the research supported in part by the National Research Foundation of South Africa (Grant No. 85768 [IFR13020116790 to GG] and Grant No. 10924 to HM). The authors also thank the following organisation for financial support: the Research Council of the University of Cape Town (staff awards to GG and HGM).

## Conflict of Interest

The authors declare that the research was conducted in the absence of any commercial or financial relationships that could be construed as a potential conflict of interest.

## Publisher’s Note

All claims expressed in this article are solely those of the authors and do not necessarily represent those of their affiliated organizations, or those of the publisher, the editors and the reviewers. Any product that may be evaluated in this article, or claim that may be made by its manufacturer, is not guaranteed or endorsed by the publisher.

## References

[1] Li J-J Shi Y Lin G-L Yang C-H Liu T-X. Genome-wide identification of neuropeptides and their receptor genes in *Bemisia tabaci* and their transcript accumulation change in response to temperature stresses. Insect Sci (2021) 28:35–46. doi: 10.1111/1744-7917.12751 31912953 PMC7818427

[2] Wang Z Zhou W Hameed MS Liu J Zeng X. Characterization and expression profiling of neuropeptides and G-protein-coupled receptors (GPCRs) for neuropeptides in the Asian citrus psyllid, *Diaphorina citri* (Hemiptera: Psyllidae). Int J Mol Sci (2018) 19:3912. doi: 10.3390/ijms19123912 30563248 PMC6321106

[3] Johnson KP Dietrich CH Friedrich F Beutel RG Wipfler B Peters RS. Phylogenomics and evolution of hemipteroid insects. Proc Natl Acad Sci USA (2018) 115:12775–80. doi: 10.1073/pnas.1815820115 PMC6294958 30478043

[4] Ghosh S Jassar O Kontsedalov S Lebedev G Wang C Turner D. A transcriptomics approach reveals putative interaction of *Candidatus* Liberibacter Solanacearum with the endoplasmic reticulum of its Psyllid vector. Insects (2019) 10(9):E279. doi: 10.3390/insects10090279 PMC6780682 31480697

[5] Misof B Liu S Meusemann K Peters RS Donath A Meyer C. Phylogenomics resolves the timing and pattern of insect evolution. Science (2014) 346:763–67. doi: 10.1126/science.1257570 25378627

[6] Jedlička P Steinbauerová V Šimek P Zahradníčková H. Functional characterization of the adipokinetic hormone in the pea aphid, *Acyrthosiphon pisum* . Comp Biochem Physiol A Mol Integr Physiol (2012) 162:51–8. doi: 10.1016/j.cbpa.2012.02.004 22357169

[7] Jedličková V Jedlička P Lee HJ. Characterization and expression analysis of adipokinetic hormone and its receptor in eusocial aphid *Pseudoregma bambucicola* . Gen Comp Endocrinol (2015) 223:38–46. doi: 10.1016/j.ygcen.2015.09.032 26432101

[8] Biello R Singh A Godfrey CJ Fernández FF Mugford ST Powell G. A chromosome-level genome assembly of the woolly apple aphid, *Eriosoma lanigerum* Hausmann (Hemiptera: Aphididae). Mol Ecol Resour (2021) 21:316–26. doi: 10.1111/1755-0998.13258 32985768

[9] Bautista MAM Egana JMC Cleofe MAS Caoili BL. Transcriptome-based *in silico* survey for putative effector genes involved in sap-feeding of the coconut scale insect, *Aspidiotus rigidus* Reyne (Hemiptera: Diaspididae). Philipp Agric Sci (2019) 102:77–89.

[10] Xue J Zhou X Zhang CX Yu LL Fan HW Wang Z. Genomes of the rice pest brown planthopper and its endosymbionts reveal complex complementary contributions for host adaptation. Genome Biol (2014) 15:521. doi: 10.1186/s13059-014-0521-0 25609551 PMC4269174

[11] Tanaka Y Suetsugu Y Yamamoto K Noda H Shinoda T. Transcriptome analysis of neuropeptides and G-protein coupled receptors (GPCRs) for neuropeptides in the brown planthopper *Nilaparvata lugens* . Peptides (2014) 53:125–33. doi: 10.1016/j.peptides.2013.07.027 23932938

[12] Zhu J Jiang F Wang X Yang P Bao Y Zhao W. Genome sequence of the small brown planthopper, *Laodelphax striatellus* . GigaScience (2017) 6:1–12. doi: 10.1093/gigascience/gix109 PMC5740986 29136191

[13] Gäde G Janssens MPE. Cicadas contain novel members of the AKH/RPCH family peptides with hypertrehalosaemic activity. Biol Chem Hoppe Seyler (1994) 375:803–09. doi: 10.1515/bchm3.1994.375.12.803 7710694

[14] Veenstra JA Hagedorn HH. Isolation of two AKH-related peptides from cicadas. Arch Insect Biochem Physiol (1995) 29:391–96. doi: 10.1002/arch.940290406 7655059

[15] Raina A Pannell L Kochansky J Jaffe H. Primary structure of a novel neuropeptide isolated from the corpora cardiaca of periodical cicadas having adipokinetic and hypertrehalosemic activities. Insect Biochem Mol Biol (1995) 25:929–32. doi: 10.1016/0965-1748(95)00032-Q 7550248

[16] Gäde G Marco HG. Flight-related metabolism and its regulatory peptides in the spittle bug *Locris arithmetica* (Cicadomorpha: Cercopidae) and the stink bugs *Nezara viridula* (Heteroptera: Pentatomidae) and *Encosternum delegorguei* (Heteroptera: Tessaratomidae). J Insect Physiol (2009) 55:1134–44. doi: 10.1016/j.jinsphys.2009.08.009 19698718

[17] Gäde G König S Marco HG. The adipokinetic hormones of Cicadomorpha (Hemiptera, Auchenorrhyncha): first steps for the development of a “green” insecticide”. In: Jenkins OP, editor. Advances in Animal Science. Chapter 4. New York: Nova Science Publishers (2021). p. 65–85.

[18] Gäde G Šimek P Marco HG. The African froghopper *Ptyelus flavescens* (suborder: Cicadomorpha) contains two novel and one known peptides of the adipokinetic hormone family: structure, function and comparison with aphid AKH (suborder: Stenorrhyncha). Amino Acids (2017) 49:1679–90. doi: 10.1007/s00726-017-2461-y 28710552

[19] Cassone BJ Wijeratne S Michel AP Stewart LR Chen Y Yan P. Virus-independent and common transcriptome responses of leafhopper vectors feeding on maize infected with semi-persistently and persistent propagatively transmitted viruses. BMC Genomics (2014) 15:133. doi: 10.1186/1471-2164-15-133 24524215 PMC3929756

[20] Nandety RS Kamita SG Hammock BD Falk BW. Sequencing and *de novo* assembly of the transcriptome of the glassy-winged sharpshooter (*Homalodisca vitripennis*). PloS One (2013) 8:e81681. doi: 10.1371/journal.pone.0081681 24339955 PMC3858241

[21] Gäde G. “Peptides of the adipokinetic hormone/red pigment-concentrating hormone family – a new take on biodiversity”. In: Vaudry H Roubos EW Coast GM Vallarino M, editors. Trends in Comparative Endocrinology and Neurobiology. Annals of the New York Academy of Science, vol. 1163. Hoboken, NJ: Wiley Blackwell (2009). p. 125–136. 10.1111/j.1749-6632.2008.03625.x 19456334

[22] Gäde G Šimek P Marco HG. A novel adipokinetic peptide in a water boatman (Heteroptera, Corixidae) and its bioanalogue in a saucer bug (Heteroptera, Naucoridae). Peptides (2007) 28:594–601. doi: 10.1016/j.peptides.2006.11.019 17215060

[23] Gäde G Šimek P Marco HG. Water scorpions (Heteroptera, Nepidae) and giant water bugs (Heteroptera, Belostomatidae): Sources of new members of the adipokinetic hormone/ red pigment-concentrating hormone family. Peptides (2007) 28:1359–67. doi: 10.1016/j.peptides.2007.05.004 17604877

[24] Gäde G Marco HG. The adipokinetic hormones of African water bugs of the Heteropteran families Nepidae and Belostomatidae. Physiol Entomol (2013) 38:279–91. doi: 10.1111/phen.12032

[25] Marco HG Šimek P Gäde G. The first decapeptide adipokinetic hormone (AKH) in Heteroptera: A novel AKH from a South African saucer bug, *Laccocoris spurcus* (Naucoridae, Laccocorinae). Peptides (2011) 32:454–60. doi: 10.1016/j.peptides.2010.10.015 20969908

[26] Gäde G Auerswald L Predel R Marco HG. Substrate usage and its regulation during flight and swimming in the backswimmer, *Notonecta glauca* . Physiol Entomol (2004) 29:84–93. doi: 10.1111/j.0307-6962.2004.0375.x

[27] Kodrík D Marco HG Šimek P Socha R Štys P Gäde G. The adipokinetic hormones of Heteroptera: a comparative study. Physiol Entomol (2010) 35:117–27. doi: 10.1111/j.1365-3032.2009.00717.x

[28] Ons S Sterkel M Diambra L Urlaub H Rivera-Pomar R. Neuropeptide precursor gene discovery in the Chagas disease vector *Rhodnius prolixus* . Insect Mol Biol (2011) 20:29–44. doi: 10.1111/j.1365-2583.2010.01050.x 20958806

[29] Marco HG Šimek P Clark KD Gäde G. Novel adipokinetic hormones in the kissing bugs *Rhodnius prolixus*, *Triatoma infestans*, *Dipetalogaster maxima* and *Panstrongylus megistus* . Peptides (2013) 41:21–30. doi: 10.1016/j.peptides.2012.09.032 23137850

[30] Predel R Neupert S Derst C Reinhardt K Wegener C. Neuropeptidomics of the bed bug *Cimex lectularius* . J Proteome Res (2018) 17:440–54. doi: 10.1021/acs.jproteome.7b00630 29148801

[31] Bailey E Field L Rawlings C King R Mohareb F Pak KH. A scaffold-level genome assembly of a minute pirate bug, *Orius laevigatus* (Hemiptera: Anthocoridae), and a comparative analysis of insecticide resistance-related gene families with hemipteran crop pests. BMC Genomics (2022) 23:45. doi: 10.1186/s12864-021-08249-y 35012450 PMC8751118

[32] Hull JJ Gross RJ Brent CS Christie AE. Filling in the gaps: A reevaluation of the *Lygus hesperus* peptidome using an expanded *de novo* assembled transcriptome and molecular cloning. Gen Comp Endocrinol (2021) 303:113708. doi: 10.1016/j.ygcen.2020.113708 33388363

[33] Liu Y Liu HW Wang HC Huang TY Liu B Yang B. *Apolygus lucorum* genome provides insights into omnivorousness and mesophyll feeding. Mol Ecol Res (2021) 21:287–300. doi: 10.1111/1755-0998.13253 32939994

[34] Gäde G Auerswald L Šimek P Marco HG Kodrík D. Red pigment-concentrating hormone is not limited to crustaceans. Biochem Biophys Res Commun (2003) 309:967–73. doi: 10.1016/j.bbrc.2003.08.107 13679068

[35] Gäde G Šimek P Marco HG. An invertebrate [hydroxyproline]-modified neuropeptide: Further evidence for a close evolutionary relationship between insect adipokinetic hormone and mammalian gonadotropin hormone family. Biochem Biophys Res Commun (2011) 414:592–97. doi: 10.1016/j.bbrc.2011.09.127 21982774

[36] Predel R Russell WK Russell DH Lopez J Esquivel J Nachman RJ. Comparative peptidomics of four related hemipteran species: pyrokinins, myosuppressin, corazonin, adipokinetic hormone, sNPF, and periviscerokinins. Peptides (2008) 29:162–67. doi: 10.1016/j.peptides.2007.08.034 18201800

[37] Gäde G Auerswald L Marco HG. Flight fuel and neuropeptidergic control of fuel mobilisation in the twig wilter, *Holopterna alata* (Hemiptera, Coreidae). J Insect Physiol (2006) 52:1171–91. doi: 10.1016/j.jinsphys.2006.08.005 17070834

[38] Gäde G Šimek P Marco HG. A sulfothreonine adipokinetic peptide – a novel post-translational modification revealed in the twig wilter *Holopterna alata* , in: Abstracts of the 27th Conference of European Comparative Endocrinologists, 25th – 29th August 2014. CECE 2014; Rennes, France; Université De Rennes (2014). p. 68.

[39] Lorenz MW Kellner R Temnow K Woodring J. Identification of the adipokinetic hormone of the large milkweed bug *Oncopeltus fasciatus* . Physiol Entomol (2008) 34:136–43. doi: 10.1111/j.1365-3032.2008.00664.x

[40] Gullan PJ Cranston PS eds. The Insects. An outline of entomology. 4th edition. Chichester: John Wiley & Sons (2010).

[41] Wheeler WC Whiting M Wheeler QD Carpenter JM. The phylogeny of the extant hexapod orders. Cladistics (2001) 17:113–69. doi: 10.1111/j.1096-0031.2001.tb00115.x 34911238

[42] Bourgoin T Campbell BC. Inferring a phylogeny for Hemiptera: Falling into the ’autapomorphic trap’. Denisia (2002) 4:67–82.

[43] Cryan JR Urban JM. Higher-level phylogeny of the insect order Hemiptera: Is Auchenorrhyncha really paraphyletic? System Entomol (2021) 37:7–21. doi: 10.1111/j.1365-3113.2011.00611.x

[44] Wang Y-H Wu H-Y Redei D Xie Q Chen Y Chen P-P. When did the ancestor of true bugs become stinky? Disentangling the phylogenomics of Hemiptera-Heteroptera. Cladistics (2017) 0:1–25. doi: 10.1111/cla.12232 34636080

[45] Blackman R Eastop V eds. Aphids on the World's crops: An identification guide. Chichester: John Wiley & Sons (2000).

[46] Elfekih S Etters P Tay WT Fumagalli M Gordon K Johnson E. Genome-wide analyses of the *Bemisia tabaci* species complex reveal contrasting patterns of admixture and complex demographic histories. PloS One (2018) 13:e0190555. doi: 10.1371/journal.pone.0190555 29364919 PMC5783331

[47] Chen W Hasegawa DK Kaur N Kliot A Pinheiro PV Luan J. The draft genome of whitefly *Bemisia tabaci* MEAM1, a global crop pest, provides novel insights into virus transmission, host adaptation, and insecticide resistance. BMC Biol (2016) 14:110. doi: 10.1186/s12915-016-0321-y 27974049 PMC5157087

[48] Xie W Chen C Yang Z Guo L Yang X Wang D. Genome sequencing of the sweetpotato whitefly *Bemisia tabaci* MED/Q. GigaScience (2017) 6:1–7. doi: 10.1093/gigascience/gix018 PMC5467035 28327996

[49] Grafton-Cardwell EE Stelinski LL Stansly PA. Biology and management of Asian citrus psyllid, vector of the Huanglongbing pathogens. Annu Rev Entomol (2013) 58:413–32. doi: 10.1146/annurev-ento-120811-153542 23317046

[50] Saha S Hosmani PS Villalobos-Ayala K Miller S Shippy T Flores M. Improved annotation of the insect vector of citrus greening disease: biocuration by a diverse genomics community. Database (Oxford) (2017) 2017:bax032. doi: 10.1093/database/bax032 29220441 PMC5502364

[51] Richards S Gibbs RA Gerardo NM Moran N Nakabachi A Stern D. Genome sequence of the pea aphid *Acyrthosiphon pisum* . PloS Biol (2010) 8:1–24. doi: 10.1371/jou PMC2826372 20186266

[52] The International Aphid Genomics Consortium. Correction: Genome sequence of the pea aphid *Acyrthosiphon pisum* . PloS Biol (2018) 16:e300029. doi: 10.1371/journal.pbio.3000029 PMC6136687 30212454

[53] Quan Q Hu X Pan B Zeng B Wu N Fang G. Draft genome of the cotton aphid *Aphis gossypii* . Insect Biochem Mol Biol (2019) 105:25–32. doi: 10.1016/j.ibmb.2018.12.007 30590189

[54] Huybrechts J Bonhomme J Minoli S Prunier-leterme N Dombrovsky A Abdel-Latief M. Neuropeptide and neurohormone precursors in the pea aphid, *Acyrthosiphon pisum* . Insect Mol Biol (2010) 2:87–95. doi: 10.1111/j.1365-2583.2009.00951.x 20482642

[55] Li C Yun X Hu X Zhang Y Sang M Liu X. Identification of G protein-coupled receptors in the pea aphid, *Acyrthosiphon pisum* . Genomics (2013) 102:345–54. doi: 10.1016/j.ygeno.2013.06.003 23792713

[56] Moran VC Zimmermann HG. Biological control of jointed cactus, *Opuntia aurantiaca* (Cactaceae), in South Africa. Agric Ecosyst Environ (1991) 37:5–27. doi: 10.1016/0167-8809(91)90136-L

[57] Sogawa K. The rice brown planthopper: Feeding physiology and host plant interactions. Annu Rev Entomol (1982) 27:49–73. doi: 10.1146/annurev.en.27.010182.000405

[58] Rashid MM Ahmed N Jahan M Islam KS Nansen C Willeeres JL. Higher fertilizer inputs increases fitness traits of brown planthopper in rice. Sci Rep (2017) 7:4719. doi: 10.1038/s41598-017-05023-7 28680158 PMC5498570

[59] Wang L Tang N Gao X Chang Z Zhang L Zhou G. Genome sequence of a rice pest, the white-backed planthopper (*Sogatella furcifera*). GigaScience (2017) 6:1–9. doi: 10.1093/gigascience/giw004 PMC5437944 28369349

[60] Burrows M. Froghopper insects leap to new heights. Nature (2003) 424:509. doi: 10.1038/424509a 12891345

[61] Gorb SN. The jumping mechanism of cicada *Cecropis vulnerata* (Auchenorrhyncha, Cercopidae): skeleton-muscle organisation, frictional surfaces, and inverse kinematic model of leg movements. Arthropod Struct Dev (2004) 33:202–20. doi: 10.1016/j.asd.2004.05.008 18089035

[62] Burrows M. Jumping performance of froghopper insects. J Exp Biol (2006) 209:4607–21. doi: 10.1242/jeb.02539 17114396

[63] Burrows M Shaw SR Sutton GP. Resilin and chitinous cuticle form a composite structure for energy storage in jumping by froghopper insects. BMC Biol (2008) 6:41. doi: 10.1186/1741-7007-6-41 18826572 PMC2584104

[64] Fisher EH Allen TC. Spittle insect damage to alfalfa and red clover. J Economic Entomol (1947) 39:821–22. doi: 10.1093/jee/39.6.821a

[65] Bodino N Cavalieri V Dongiovanni C Saladini MA Simonetto A Volani S. Spittle bugs of Mediterranean olive groves: Host-plant exploitation throughout the year. Insects (2020) 11:130. doi: 10.3390/insects11020130 32085449 PMC7074542

[66] Beyer BA Srinivasan R Roberts PM Abney MR. Biology and management of the three-cornered alfalfa hopper (Hemiptera: Membracidae) in alfalfa, soybean, and peanut. J Integr Pest Managem (2017) 8:1–10. doi: 10.1093/jipm/pmx003

[67] Špryňar P. First records of the rhododendron leafhopper (*Graphocephala fennahi*) (Hemiptera: Auchenorrhyncha: Cicadellidae) from the Czech Republic. Plant Protec Sci (2005) 41:38–41. doi: 10.17221/2731-PPS

[68] Ma J-Z Lu H-Y Li X-S Tian Y. Interfacial phenomena of water striders on water surfaces: a review from biology to biomechanics. Zool Res (2020) 41:231–46. doi: 10.24272/j.issn.2095-8137.2020.029 PMC7231474 32212429

[69] Armisén D Rajakumar R Friedrich M Benoit JB Robertson HM Panfilio KA. The genome of the water strider *Gerris buenoi* reveals expansions of gene repertoires associated with adaptations to life on the water. BMC Genomics (2018) 19:832. doi: 10.1186/s12864-018-5163-2 30463532 PMC6249893

[70] Ye Z Damgard J Yang H Hebsgaard MB Weir T Bu W. Phylogeny and diversification of the true water bugs (Insecta: Hemiptera: Heteroptera: Nepomorpha). Cladistics (2019) 36:72–87. doi: 10.1111/cla.12383 34618947

[71] Balmert A Bohn HF Ditschke-Kuru P Barthlott W. Dry under water: Comparative morphology and functional aspects of air-retaining insect surfaces. J Morphol (2011) 272:442–51. doi: 10.1002/jmor.10921 21290417

[72] Seymour RS Jones KK Hetz SK. Respiratory function of the plastron in the aquatic bug *Aphelocheirus aestivalis* (Hemiptera, Aphelocheiridae). J Exp Biol (2015) 218:2840–46. doi: 10.1242/jeb.125328 26206357

[73] Mesquita RD Vionette-Amaral RJ Lowenberger C Rivera-Pomar R Monteiro FA Minx P. Genome of *Rhodnius prolixus*, an insect vector of Chagas disease, reveals unique adaptations to hematophagy and parasite infection. Proc Natl Acad Sci USA (2015) 112:14936–41. doi: 10.1073/pnas.1506226112 PMC4672799 26627243

[74] Reinhardt K Siva-Jothy MT. Biology of the bed bugs (Cimicidae). Annu Rev Entomol (2007) 52:351–74. doi: 10.1146/annurev.ento.52.040306.133913 16968204

[75] Benoit JB Adelman ZN Reinhardt K Dolan A Poelchau M Jenings EC. Unique features of a global human ectoparasite identified through sequencing of the bed bug genome. Nat Comm (2016) 7:10165. doi: 10.1038/ncomms10165 PMC4740739 26836814

[76] Rosenfeld JA Reeves D Brugler MR Narechania A Simon S Durrett R. Genome assembly and geospatial phylogenomics of the bed bug *Cimex lectularius* . Nat Comm (2016) 7:10164. doi: 10.1038/ncomms10164 PMC4740774 26836631

[77] Scott DR. An annotated listing of host plants of *Lygus hesperus* Knight. Bull Entomol Soc Am (1977) 23:19–22. doi: 10.1093/besa/23.1.19

[78] Ferguson KB Visser S Dalikova M Provaznikova I Urbaneja A Perez-Hedo M. Jekyll or Hyde? The genome (and more) of *Nesidiocoris tenuis*, a zoophytophagous predatory bug that is both a biological control agent and a pest. Insect Mol Biol (2021) 30:188–209. doi: 10.1111/imb.12688 33305885 PMC8048687

[79] Bai Y Shi Z Zhou W Wang G Shi X He K. Chromosome-level genome assembly of the mirid predator *Cyrtorhinus lividipennis* Reuter (Hemiptera: Miridae), an important natural enemy in the rice ecosystem. Mol Ecol Res (2021) 22:1086–1099. doi: 10.1111/1755-0998.13516 34581510

[80] Yang B. Genome sequencing of *Pachypeltis micranthus* Mu et Liu (Hemiptera: Miridae), a potential biological control agent for *Mikania micrantha* . Dryad Digital Repository (2021). doi: 10.5061/dryad.0k6djhb18

[81] Dzerefos CM Witkowski ETF Toms R. Life-history traits of the edible stinkbug, *Encosternum delegorguei* (Hem., Tessaratomidae), a traditional food in southern Africa. J Appl Entomol (2009) 133:739–59. doi: 10.1111/j.1439-0418.2009.01425.x

[82] Crowley L Barclay MVL. The genome sequence of the bishop’s mitre shieldbug, *Aelia acuminata* (Linnaeus, 1758) [version 1; peer review: 1 approved]. Wellcome Open Res (2021) 6:320. doi: 10.12688/wellcomeopenres.17400.1 35187268 PMC8817068

[83] Sparks ME Bansal R Benoit JB Blackburn MB Chao H Chen M. Brown marmorated stink bug, *Halyomorpha halys* (Stål), genome: putative underpinnings of polyphagy, insecticide resistance potential and biology of a top worldwide pest. BMC Genomics (2020) 21:227. doi: 10.1186/s12864-020-6510-7 32171258 PMC7071726

[84] Lavore A Perez-Gianmarco L Esponda-Behrens N Palacio V Catalano MI Rivera-Pomar R. *Nezara viridula* (Hemiptera: Pentatomidae) transcriptomic analysis and neuropeptidomics. Sci Rep (2018) 8:17244. doi: 10.1038/s41598-018-35386-4 30467353 PMC6250713

[85] Dalla S Dobler S. Gene duplications circumvent trade-offs in enzyme function: Insect adaptation to toxic host plants. Evolution (2016) 70:2669–920. doi: 10.1111/evo.13077 27683239

[86] Groeters FR Dingle H. Genetic and maternal influences on life history plasticity in response to photoperiod by milkweed bugs (*Oncopeltus fasciatus*). Am Nat (1987) 129:3332–46. doi: 10.1086/284640

[87] Panfilio KA Vargas Jentzsch IM Benoit JB Erezyilmaz D Suzuki Y Colella S. Molecular evolutionary trends and feeding ecology diversification in the Hemiptera, anchored by the milkweed bug genome. Genome Biol (2019) 20:64. doi: 10.1186/s13059-019-1660-0 30935422 PMC6444547

[88] Dudley R. The evolutionary physiology of animal flight: paleobiological and present perspectives. Annu Rev Physiol (2000) 62:135–55. doi: 10.1146/annurev.physiol.62.1.135 10845087

[89] Marco HG Šimek P Gäde G. Unique members of the adipokinetic hormone family in butterflies and moths (Insecta, Lepidoptera). Front Physiol (2020) 11:614552. doi: 10.3389/fphys.2020.614552 33391031 PMC7773649

[90] Kennedy JS. The migration of the desert locust (*Schistocerca gregaria* Forsk.) I. The behaviour of swarms. II. A theory of long-range migrations. Phil Trans R Soc B (1951) 235:163–290. doi: 10.1098/rstb.1951.0003 24541037

[91] Agrawal AA. Monarchs and milkweed: a migrating butterfly, a poisonous plant, and their remarkable story of coevolution. Princeton: Princeton University Press (2017). 283 p. Available at: https://lccn.loc.gov/2016034053.

[92] Kisimoto R Rosenberg LJ. “Long-distance migration in delphacid planthoppers”. In: Denno RF Perfect TJ, editors. Planthoppers: Their Ecology and Management. New York: Chapman and Hall (1994). p. 302–22.

[93] Antolinez CA Moyneur T Martini X Rivera MJ. High temperatures decrease the flight capacity of *Diaphorina citri* Kuwayama (Hemiptera: Liviidae). Insects (2021) 12:394. doi: 10.3390/insects12050394 33946666 PMC8145625

[94] Zhang Y Wang L Wu K Wyckhuis KAG Heimpel GE. Flight performance of the soybean aphid, *Aphis glycines* (Hemiptera: Aphididae) under different temperatures and humidity regimens. Environ Entomol (2008) 37:301–6. doi: 10.1093/ee/37.2.301 18419900

[95] Minter M Pearson A Lim KS Wilson K Chapman JW Jones CM. The tethered flight technique as a tool for studying life-history strategies associated with migration in insects. Ecol Entomol (2018) 43:397–411. doi: 10.1111/een.12521 30046219 PMC6055614

[96] Popham EJ. The migration of aquatic bugs with special reference to the Corixidae (Hemiptera Heteroptera). Arch Hydrobiol (1964) 60:450–96.

[97] Duviard D. Flight activity of Belostomatidae in central Ivory Coast. Oecologia (1974) 15:321–28. doi: 10.1007/BF00345429 28308627

[98] Stewart SD Gaylor MJ. Effect of age, sex, and reproductive status on flight by the tarnished plant bug (Heteroptera: Miridae). Environ Entomol (1994) 23:80–4. doi: 10.1093/ee/23.1.80

[99] Blackmer J Naranjo SE Williams LH. Tethered and untethered flight by *Lygus hesperus* and *Lygus lineolaris* (Heteroptera: Miridae). Environ Entomol (2004) 33:1389–400. doi: 10.1603/0046-225X-33.5.1389

[100] Wiman NG Walton VM Shearer PW Rondon SI Lee JC. Factors affecting flight capacity of brown marmorated stink bug, *Halyomorpha halys* (Hemiptera: Pentatomidae). J Pest Sci (2015) 8:37–47. doi: 10.1007/s10340-014-0582-6

[101] Aita RC Kees AM Aukema BH Hutchison WD Koch RL. Effects of starvation, age, and mating status on flight capacity of laboratory-reared brown marmorated stink bug (Hemiptera: Pentatomidae). Environ Entomol (2021) 50:532–40. doi: 10.1093/ee/nvab019 33822022

[102] Todd JW. Ecology and behavior of *Nezara viridula* . Annu Rev Entomol (1989) 34:273–92. doi: 10.1146/annurev.en.34.010189.001421

[103] Gu H Walter GH. Flight of green vegetable bugs *Nezara viridula* (L.) in relation to environmental variables. J Appl Entomol (1989) 108:347–54. doi: 10.1111/j.1439-0418.1989.tb00467.x

[104] Dingle H. Some factors affecting flight activity in individual milkweed bugs (*Oncopeltus*). J Exp Biol (1966) 44:335–43. doi: 10.1242/jeb.44.2.335 5957027

[105] Akhoundi M Sereno D Durand R Mirzaei A Bruel C Delauny P. Bed bugs (Hemiptera, Cimicidae): Overview of classification, evolution and dispersion. Int J Environ Res Public Health (2020) 17:4576. doi: 10.3390/ijerph17124576 32630433 PMC7345932

[106] Socha R. *Pyrrhocoris apterus* (Heteroptera) –an experimental model species: A review. Eur J Entomol (1993) 90:241–86.

[107] Kammer AE Heinrich B. Insect flight metabolism. Adv Insect Physiol (1978) 13:133–228. doi: 10.1016/S0065-2806(08)60266-0

[108] Gäde G. The hormonal integration of insect flight metabolism. Zool JB Physiol (1992) 96:211–25.

[109] Stone JV Mordue W Batley KE Morris HR. Structure of locust adipokinetic hormone, a neurohormone that regulates lipid utilisation during flight. Nature (1976) 263:207–11. doi: 10.1038/263207a0 958472

[110] Bradfield JY Keeley LL. Adipokinetic hormone gene sequence from *Manduca sexta* . J Biol Chem (1989) 264:12791–93. doi: 10.1016/S0021-9258(18)51555-6 2753887

[111] Marco HG Gäde G. “Adipokinetic hormone: a hormone for all seasons?” In: Saleuddin S Lange AB Orchard I, editors. Advances in Invertebrate (Neuro)Endocrinology: A Collection of Reviews in the Post-Genomic Era, Vol. 2. New Jersey: Apple Academic Press (2020). p.p 126–70.

[112] Staubli F Jorgensen TJ Cazzamali G Williamson M Lenz C Sondergaard L. Molecular identification of the insect adipokinetic hormone receptors. Proc Natl Acad Sci USA (2002) 99:3446–51. doi: 10.1073/pnas.052556499 PMC122543 11904407

[113] Park Y Kim YJ Adams ME. Identification of G protein-coupled receptors for *Drosophila* Prxamide peptides, CCAP, corazonin, and AKH supports a theory of ligand-receptor coevolution. Proc Natl Acad Sci USA (2002) 99:11423–28. doi: 10.1073/pnas.162276199 PMC123272 12177421

[114] Gäde G Auerswald L. Mode of action of neuropeptides from the adipokinetic hormone family. Gen Comp Endocrinol (2003) 132:10–20. doi: 10.1016/S0016-6480(03)00159-X 12765639

[115] Van der Horst D Ryan RO. “Lipid Transport”. In: Reference Module in Life Sciences. Amsterdam: Elsevier (2017). doi: 10.1016/B978-0-12-809633-8.04045-0

[116] Arrese EL Canavoso LE Jouni ZE Pennington JE Tsuchida K Wells MA. Lipid storage and mobilization in insects: current status and future directions. Insect Biochem Mol Biol (2001) 31:7–17. doi: 10.1016/S0965-1748(00)00102-8 11102830

[117] Auerswald L Siegert KJ Gäde G. Activation of triacylglycerol lipase in the fat body of a beetle by adipokinetic hormone. Insect Biochem Mol Biol (2005) 35:461–70. doi: 10.1016/j.ibmb.2005.01.010 15804579

[118] Toprak U. The role of peptide hormones in insect lipid metabolism. Front Physiol (2020) 11:434. doi: 10.3389/fphys.2020.00434 32457651 PMC7221030

[119] Oliveira GA Baptista DL Guimarães-Motta H Almeida IC Masuda H Atella GC. Flight-oogenesis syndrome in a blood-sucking bug: Biochemical aspects of lipid metabolism. Arch Insect Biochem Physiol (2006) 62:164–75. doi: 10.1002/arch.20132 16933278

[120] Gäde G. “Extraction, purification and sequencing of adipokinetic/red pigment-concentrating hormone family peptides”. In: McCaffery AR Wilson ID, editors. Chromatography and Isolation of Insect Hormones and Pheromones. New York: Plenum Press (1990). p. 165–82.

[121] Socha R Kodrík D Šimek P Patočková M. The kind of AKH-mobilized energy substrates in insects can be predicted without a knowledge of the hormone structure. Eur J Entomol (2004) 101:29–35. doi: 10.14411/eje.2004.007

[122] Ashurst DE Luke BM. Lipid inclusions in the flight muscles of belostomatid water-bugs. Zeitschr Zellforsch Mikroskop Anat (1968) 92:270–74. doi: 10.1007/BF00335652 4190621

[123] Ward JP Candy DJ Smith SN. Lipid storage and changes during flight by triatomine bugs (*Rhodnius prolixus* and *Triatoma infestans)* . J Insect Physiol (1982) 28:527–34. doi: 10.1016/0022-1910(82)90033-6

[124] König S Bayer M Marco H Gäde G. The hypertrehalosaemic neuropeptide conformational twins of cicadas consist of only L−amino acids: are they *cis*–*trans* isomers? Amino Acids (2019) 51:1023–28. doi: 10.1007/s00726-019-02742-1 31073692

[125] Gäde G Šimek P. A novel member of the adipokinetic peptide family in a “living fossil”, the ice crawler *Galloisiana yuasai*, is the first identified neuropeptide from the order Grylloblattodea. Peptides (2010) 31:372–76. doi: 10.1016/j.peptides.2009.10.016 19857536

[126] Byrne DN. Migration and dispersal by the sweet potato whitefly, *Bemisia tabaci* . Agricult For Meteor (1999) 97:309–16. doi: 10.1016/S0168-1923(99)00074-X 28307104

[127] Gäde G Šimek P Marco HG. Identification and bioactivity evaluation of the first neuropeptide from the lesser-known insect order Embioptera (webspinner). Amino Acids (2016) 48:1677–84. doi: 10.1007/s00726-016-2229-9 27074720

[128] Cockbain AJ. Fuel utilization and duration of tethered flight in *Aphis fabae* Scop. J Exp Biol (1961) 38:163–74. doi: 10.1242/jeb.38.1.163

[129] Liquido NJ Irwin ME. Longevity, fecundity, change in degree of gravidity and lipid content with adult age, and lipid utilisation during tethered flight of alates of the corn leaf aphid, *Rhopalosiphum maidis* . Ann Appl Bio (1986) 108:449–59. doi: 10.1111/j.1744-7348.1986.tb01984.x

[130] Gäde G Marco HG Šimek P Marais E. The newly discovered insect order Mantophasmatodea contains a novel member of the adipokinetic hormone family of peptides. Biochem Biophys Res Commun (2005) 330:598–603. doi: 10.1016/j.bbrc.2005.02.185 15796925

[131] Gäde G Šimek P Marco HG. Structural diversity of adipokinetic hormones in the hyperdiverse coleopteran Cucujiformia. Arch Insect Biochem Physiol (2019) 102:e21611. doi: 10.1002/arch.21611 31471923

[132] Chen R. Studies on lipids as fuel of flight in the brown planthopper (*Nilaparvata lugens* Stål). Acta Entomol Sin (1983) 26:42–9.

[133] Padgham DE. Flight fuels in the brown planthopper, *Nilaparvata lugens* . J Insect Physiol (1983) 29:95–9. doi: 10.1016/0022-1910(83)90111-7

[134] Lu K Zhang X Chen X Li Y Li W Cheng Y. Adipokinetic hormone receptor mediates lipid mobilization to regulate starvation resistance in the brown planthopper, *Nilaparvata lugens* . Front Physiol (2018) 9:1730. doi: 10.3389/fphys.2018.01730 30555355 PMC6281999

[135] Lu K Wang Y Chen X Zhang X Li W Cheng Y. Adipokinetic hormone receptor mediates trehalose homeostasis to promote vitellogenin uptake by oocytes in *Nilaparvata lugens* . Front Physiol (2019) 9:1904. doi: 10.3389/fphys.2018.01904 30687120 PMC6338042

[136] Gäde G Marco HG. The adipokinetic hormone of Mantodea in comparison to other Dictyoptera. Arch Insect Biochem Physiol (2017) 94:e21376. doi: 10.1002/arch.21376 28225562

[137] Gäde G Marco HG. The adipokinetic hormone (AKH) of one of the most basal orders of Pterygota: structure and function of Ephemeroptera AKH. J Insect Physiol (2012) 58:1390–96. doi: 10.1016/j.jinsphys.2012.08.001 22885738

[138] Zhou L Hoy CW Miller SA Nault LR. Marking methods and field experiments to estimate aster leafhopper (*Macrosteles quadrilineaus*) dispersal rates. Environ Entomol (2003) 32:1177–86. doi: 10.1603/0046-225X-32.5.1177

[139] Riley J Downham M Cooter R. Comparison of the performance of *Cicadulina* leafhoppers on flight mills with that to be expected in free flight. Entomol Exp Appl (1997) 83:317–22. doi: 10.1046/j.1570-7458.1997.00186.x

[140] Gorb SN Perez Goodwyn PJ. Wing-locking mechanisms in aquatic Heteroptera. J Morph (2003) 257:127–46. doi: 10.1002/jmor.10070 12833375

[141] Kaitala A Hulden L. Significance of spring migration and flexibility in flight-muscle histolysis in waterstriders (Heteroptera, Gerridae). Ecol Entomol (1990) 15:409–18. doi: 10.1111/j.1365-2311.1990.tb00824.x

[142] Lee RF Polhemus JT Cheng L. Lipids of the water-strider *Gerris remigis* Say (Heteroptera: Gerridae). Seasonal and developmental variations. Comp Biochem Physiol B (1975) 51:451–56. doi: 10.1016/0305-0491(75)90037-1 1149432

[143] Csabai Z Boda P Bernath B Kriska G Horvath G. A ‘polarisation sun-dial’ dictates the optimal time of day for dispersal by flying aquatic insects. Freshw Biol (2006) 51:1341–50. doi: 10.1111/j.1365-2427.2006.01576.x

[144] Walton GA. Field experiments on the flight of *Notonecta maculata* Fabr. (Hemipt.). Trans Soc Brit Entomol (1935) 2:137–44.

[145] Gäde G Šimek P Marco HG. The adipokinetic peptides in Diptera: Structure, function, and evolutionary trends. Front Endocrinol (2020) 11:153. doi: 10.3389/fendo.2020.00153 PMC7136388 32296388

[146] Canavoso LE Rubiolo ER. Interconversion of lipophorin particles by adipokinetic hormone in the hemolymph of *Panstrongylus megistus*, *Dipetalogaster maximus*, and *Triatoma infestans* (Hemiptera: Reduviidae). Comp Biochem Physiol (1995) 112A:143–50. doi: 10.1016/0300-9629(95)00077-K

[147] Goldsworthy GJ Mordue W. Adipokinetic hormones: functions and structures. Biol Bull (1989) 177:218–24. doi: 10.2307/1541936

[148] Canavoso LE Stariolo R Rubiolo ER. Flight metabolism in *Panstrongylus megistus* (Hemiptera: Reduviidae): the role of carbohydrates and lipids. Mem Inst Oswaldo Cruz (2003) 98:909–14. doi: 10.1590/S0074-02762003000700009 14762517

[149] Patel H Orchard I Veenstra JA Lange AB. The distribution and physiological effects of three evolutionarily and sequence-related neuropeptides in *Rhodnius prolixus*: Adipokinetic hormone, corazonin and adipokinetic hormone/corazonin-related peptide. Gen Comp Endocrinol (2014) 195:1–8. doi: 10.1016/j.ygcen.2013.10.012 24184870

[150] Zandawala M Hamoudi Z Lange AB Orchard I. Adipokinetic hormone signalling system in the Chagas disease vector, *Rhodnius prolixus* . Insect Mol Biol (2015) 24:264–76. doi: 10.1111/imb.12157 25545120

[151] Alves-Bezerra M de Paula IF Medina JM Silva-Oliveira G Medeiros JS Gäde G. Adipokinetic hormone receptor gene identification and its role in triacylglycerol metabolism in the blood-sucking insect *Rhodnius prolixus* . Insect Biochem Mol Biol (2016) 69:51–60. doi: 10.1016/j.ibmb.2015.06.013 26163435

[152] Ons S Lavore A Sterkel M Wulff JP Sierra I Martínez-Barnetche J. Identification of G protein coupled receptors for opsines and neurohormones in *Rhodnius prolixus*. Genomic and transcriptomic analysis. Insect Biochem Mol Biol (2016) 69:34–50. doi: 10.1016/j.ibmb.2015.05.003 25976540

[153] Ibrahim E Hejníková M Shail HA Doležel D Kodrík D. Adipokinetic hormone activities in insect body infected by entomopathogenic nematode. J Insect Physiol (2017) 98:347–55. doi: 10.1016/j.jinsphys.2017.02.009 28254268

[154] Leyria J El-Mawed H Orchard I Lange AB. Regulation of a trehalose-specific facilitated transporter (TRET) by insulin and adipokinetic hormone in *Rhodnius prolixus*, a vector of Chagas disease. Front Physiol (2021) 12:624165. doi: 10.3389/fphys.2021.624165 33643069 PMC7902789

[155] Smith CR Smith CD Robertson HM Helmkampf M Zimin A Yandell M. Draft genome of the red harvester ant *Pogonomyrmex barbatus* . Proc Natl Acad Sci USA (2011) 108:5667–72. doi: 10.1073/pnas.1007901108 PMC3078412 21282651

[156] Veenstra JA Rodriguez L Weaver RJ. Allatotropin, leukokinin and AKH in honey bees and other Hymenoptera. Peptides (2012) 35:122–30. doi: 10.1016/j.peptides.2012.02.019 22406227

[157] Lu Y Wu K Guo Y. Flight potential of *Lygus lucorum* (Meyer-Dür) (Heteroptera: Miridae). Environ Entomol (2007) 36:1007–13. doi: 10.1093/ee/36.5.1007 18284721

[158] Gäde G Marco HG. The decapod red pigment-concentrating hormone (Panbo-RPCH) is the first identified neuropeptide of the order Plecoptera and is interpreted as homoplastic character state. Gen Comp Endocrinol (2015) 221:228–35. doi: 10.1016/j.ygcen.2015.02.011 25733206

[159] Goldsworthy GJ. The endocrine control of flight metabolism in locusts. Adv Insect Physiol (1983) 17:149–204. doi: 10.1016/S0065-2806(08)60218-0

[160] Kodrík D Socha R Šimek P Zemek R Goldsworthy G. A new member of the AKH/RPCH family that stimulates locomotory activity in the firebug, *Pyrrhocoris apterus* (Heteroptera). Insect Biochem Mol Biol (2000) 30:489–98. doi: 10.1016/S0965-1748(00)00025-4 10802240

[161] Kodrík D Šimek P Lepŝa L Socha R. Identification of the cockroach neuropeptide Pea-CAH-II as a second adipokinetic hormone in the firebug *Pyrrhocoris apterus* . Peptides (2002) 23:583–85. doi: 10.1016/S0196-9781(01)00627-1 11836011

[162] Bártů I Tomčala A Socha R Šimek P Kodrík D. Analysis of the lipids mobilized by adipokinetic hormones in the firebug *Pyrrhocoris apterus* (Heteroptera: Pyrrhocoridae). Eur J Entomol (2010) 107:509–20. doi: 10.14411/eje.2010.058

[163] Tomčala A Bártů I Šimek P Kodrík D. Locust adipokinetic hormones mobilize diacylglycerols selectively. Comp Biochem Physiol B (2010) 156:26–32. doi: 10.1016/j.cbpb.2010.01.015 20139028

[164] Goldsworthy GJ Kodrík D Comley R Lightfoot M. A quantitative study of the adipokinetic hormone of the firebug, *Pyrrhocoris apterus* . J Insect Physiol (2002) 48:1103–09. doi: 10.1016/S0022-1910(02)00203-2 12770033

[165] Kodrík D Socha R Syrová Z. Developmental and diel changes of adipokinetic hormone in CNS and haemolymph of the flightless wing-polymorphic bug, *Pyrrhocoris apterus* . J Insect Physiol (2003) 49:53–61. doi: 10.1016/S0022-1910(02)00245-7 12770016

[166] Kodrík D Socha R. The effect of insecticide on adipokinetic hormone titre in the insect body. Pest Manag Sci (2005) 61:1077–82. doi: 10.1002/ps.1087 15966049

[167] Kodrík D Bártů I Socha R. Adipokinetic hormone (Pyrap-AKH) enhances the effect of a pyrethroid insecticide against the firebug *Pyrrhocoris apterus* . Pest Manag Sci (2009) 66:425–31. doi: 10.1002/ps.1894 20013955

[168] Kodrík D Stašková T Jedličková V Weyda F Závodská R Pflegerová J. Molecular characterization, tissue distribution, and ultrastructural localization of adipokinetic hormones in the CNS of the firebug *Pyrrhocoris apterus* (Heteroptera, Insecta). Gen Comp Endocrinol (2015) 210:1–11. doi: 10.1016/j.ygcen.2014.10.014 25449136

[169] Marchal E Schellens S Monjon E Bruyninckx E Marco HG Gäde G. Analysis of peptide ligand specificity of different insect adipokinetic hormone receptors. Int J Mol Sci (2018) 19:542. doi: 10.3390/ijms19020542 29439466 PMC5855764

